# SETD5 modulates homeostasis of hematopoietic stem cells by mediating RNA Polymerase II pausing in cooperation with HCF-1

**DOI:** 10.1038/s41375-021-01481-1

**Published:** 2021-12-01

**Authors:** Mengke Li, Chen Qiu, Yujie Bian, Deyang Shi, Bichen Wang, Qiuyi Ma, Xiaomin Wang, Jun Shi, Lianfeng Zhang, Yuanwu Ma, Ping Zhu, Tao Cheng, Yajing Chu, Weiping Yuan

**Affiliations:** 1 State Key Laboratory of Experimental Hematology, National Clinical Research Center for Blood Diseases, Institute of Hematology & Blood Diseases Hospital, Chinese Academy of Medical Sciences and Peking Union Medical College, Tianjin, China; 2 Key Laboratory of Human Disease Comparative Medicine, National Health Commission of China (NHC), Beijing Engineering Research Center for Experimental Animal Models of Human Critical Diseases, Institute of Laboratory Animal Science, Chinese Academy of Medical Sciences, Peking Union Medicine College, Beijing, China

**Keywords:** Haematopoietic stem cells, Cell biology

## Abstract

*SETD5* mutations were identified as the genetic causes of neurodevelopmental disorders. While the whole-body knockout of *Setd5* in mice leads to embryonic lethality, the role of SETD5 in adult stem cell remains unexplored. Here, a critical role of *Setd5* in hematopoietic stem cells (HSCs) is identified. Specific deletion of *Setd5* in hematopoietic system significantly increased the number of immunophenotypic HSCs by promoting HSC proliferation. *Setd5*-deficient HSCs exhibited impaired long-term self-renewal capacity and multiple-lineage differentiation potentials under transplantation pressure. Transcriptome analysis of *Setd5*-deficient HSCs revealed a disruption of quiescence state of long-term HSCs, a cause of the exhaustion of functional HSCs. Mechanistically, SETD5 was shown to regulate HSC quiescence by mediating the release of promoter-proximal paused RNA polymerase II (Pol II) on E2F targets in cooperation with HCF-1 and PAF1 complex. Taken together, these findings reveal an essential role of SETD5 in regulating Pol II pausing-mediated maintenance of adult stem cells.

## Introduction

Stem cells maintain their self-renewal and differentiation through tight regulation of gene expression patterns at the transcriptional and epigenetic levels. SETD5 is a member of SET domain-containing histone lysine methyltransferases (KMTs) family [1, 2]. Like its orthologs including yeast Set3p and *Drosophila* UpSET [3, 4], SETD5 has been shown to lack methyltransferase activity in several studies [5–7]. Interestingly, *de novo* mutations in the *Setd5* gene have been identified as the genetic causes of intellectual disability and autism spectrum disorders [8–11]. More recently, SETD5 was identified as a key mediator of pancreatic cancer resistance to MEK1/2 inhibition targeted therapy [7], suggesting that its physiological and pathological roles might be diverse and tissue or cell type specific.

*Setd5* is broadly expressed in mouse tissues. Deletion of *Setd5* resulted in embryonic lethality at E10.5-11.5 [5], while *Setd5* haploinsufficiency led to disrupted developmental gene expression and cognition [6]. Interestingly, in the placenta of *Setd5*-deficient mice, only maternal-derived mature red blood cells but not embryo-derived nucleated red blood cells were observed. In addition, the number of CD41^+^ early hematopoietic cells declined in the blood island of *Setd5* homozygous knockout mice [5]. These data suggested a potential role of SETD5 in hematopoiesis.

Hematopoietic stem and progenitor cells (HSPCs) maintain hematopoietic homeostasis through well-orchestrated balance between self-renewal and differentiation. The majority of HSCs exist in a quiescent state to prevent HSC exhaustion [12, 13]. Transcriptionally engaged RNA Pol II experiences a temporary stalling after initiation on many metazoan genes and was considered as an important regulatory process in gene transcription [14, 15]. Despite it was reported that Pol II pausing modulates hematopoietic stem cell generation in zebrafish [16], the in vivo effect of Pol II pausing on the maintenance of HSCs in mammals remains to be further elucidated.

Here in this study, we explored the role of *Setd5* in hematopoiesis and found that depletion of *Setd5* in hematopoietic system significantly increased immune-phenotypically defined HSCs with impaired HSC quiescence at the single cell level. Mechanistic studies revealed a novel transcriptional mechanism of SETD5 in governing HSC quiescence by Pol II pausing-mediated regulation of cell cycle activating genes.

## Materials and methods

### Animals

*Setd5* floxed (*Setd5*^*fl/fl*^) mice were generated by inserting loxp sites flanking exons 3-6, which when deleted, would result in a frame-shift and form a premature stop codon in the reading frame. To induce *Mx1-Cre* expression, adult mice were intraperitoneally injected (ip) with 10 μg/g polyinosinic-polycytidylic acid (pIpC, InvivoGen) 3 times every 48 h. All primers and antibodies used in this study are listed in Supplemental Table 1. All animal research was approved by the Institutional Animal Care and Use Committee of the State Key Laboratory of Experimental Hematology.

### Additional methods

Additional methods are provided in Supplemental Information.

## Results

### Genetic ablation of *Setd5* causes phenotypic HSPC expansion

**Fig. 1:**
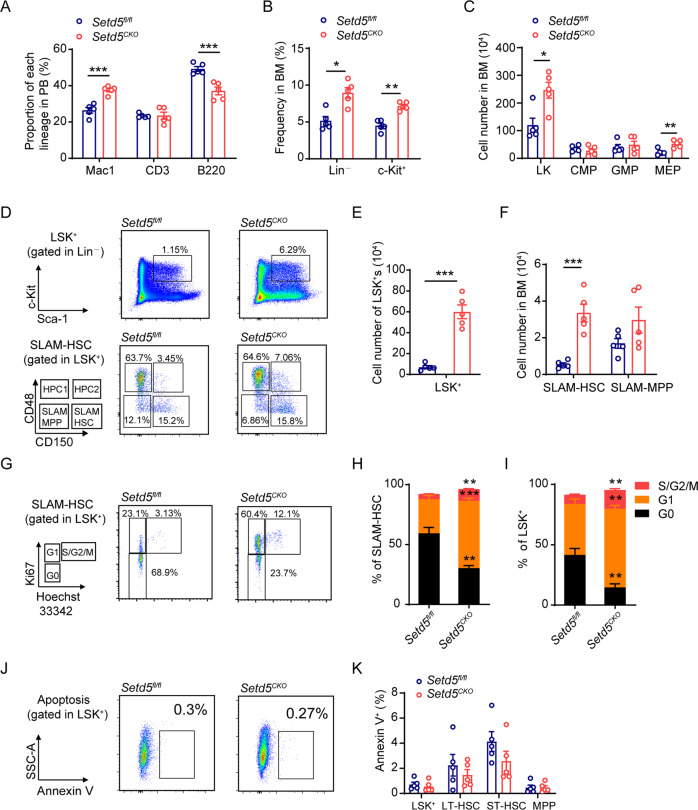
Setd5 deficiency causes phenotypic HSPC expansion. **A** FACS analysis of T, B, and myeloid cells frequency in PB cells; *n* = 5. **B** Relative frequency of immature cells (Lin^–^ and c-Kit^+^) in BM. Lin: lineage cocktail; *n* = 5. **C** The absolute cell number of HPC (LK: Lin^–^c-Kit^+^Sca-1^–^), CMP (Lin^–^c-Kit^+^Sca1^–^CD34^+^CD16/32^low^), GMP (Lin^–^c-Kit^+^Sca1^–^CD34^+^CD16/32^high^) and MEP (Lin^–^c-Kit^+^Sca1^–^CD34^–^CD16/32^low^) populations in *Setd5*^*fl/fl*^ and *Setd5*^*CKO*^ mice; n = 5. **D**–**F** FACS analysis of LSK^+^s (Lin^–^Sca1^+^c-Kit^+^) and SLAM-HSCs (Lin^–^Sca1^+^c-Kit^+^CD150^+^CD48^–^) and absolute cell number in BM. SLAM-MPP: Lin^–^Sca1^+^c-Kit^+^CD150^–^CD48^–^, HPC1: Lin^–^Sca1^+^c-Kit^+^CD150^–^CD48^+^, HPC2: Lin^–^Sca1^+^c-Kit^+^CD150^+^CD48^+^; n = 5. **G** Representative FACS profiles of Ki67 staining of SLAM-HSCs. **H**, **I** The frequencies of G0, G1, S/G2/M phases in SLAM-HSCs and LSK^+^s are shown; *n* = 4. **J**, **K** Apoptosis analysis of HSPCs in *Setd5*^*fl/fl*^ and *Setd5*^*CKO*^ mice, LT-HSC: Lin^–^Sca1^+^c-Kit^+^CD34^–^Flt3^low^, ST-HSC: Lin^–^Sca1^+^c-Kit^+^CD34^+^Flt3^low^, MPP: Lin^–^Sca1^+^c-Kit^+^CD34^+^Flt3^+^; *n* = 5. Data are represented as mean ± SEM. * *P* < 0.05, ** *P* < 0.01, *** *P* < 0.001.

To investigate the biological roles of *Setd5* in hematopoietic system, we generated *Vav-Cre*;*Setd5*^*fl/fl*^ (*Setd5*^*CKO*^) mice and confirmed the deletion efficiency of *Setd5* (Figure S1A-E). *Setd5*^*CKO*^ mice had slightly higher monocyte count in peripheral blood (PB) and increased spleen (SP) weight but with comparable cellularity than those of the controls (Figure S1F-H). We observed modest decreased frequencies of B and T lymphocytes in the PB and bone marrow (BM) and a significant increased frequency of granulocytes/monocytes in PB of *Setd5*^*CKO*^ mice when compared with the control animals (Fig. 1A, Figure S1I).

Examination of the hematopoietic progenitor cells (HPCs, LK) revealed that the relative frequency of Lin^–^ and c-Kit^+^ cells was significantly increased in the BM of *Setd5*^*CKO*^ mice (Fig. 1B). The cell number of LKs and megakaryocyte–erythroid progenitors (MEP) in the BM was also increased, while common myeloid progenitors (CMP) and granulocyte-monocyte progenitors (GMP) remained unchanged (Fig. 1C, Figure S2A). We also determined the cell number of common lymphoid progenitors (CLPs), which showed no difference between the two groups (Figure S2B-C). Colony forming assay revealed that the frequencies of the functional HPCs remained unchanged with *Setd5* deletion (Figure S2D).

We further examined the effect of *Setd5* deficiency at HSC levels. CD150 and CD48 [17] were employed to evaluate SLAM-HSCs. LSK^+^, SLAM-HSC, HPC1 and HPC2 showed a dramatic increase in the cell number by ~5 to ~10 fold in *Setd5*^*CKO*^ mice (Fig. 1D-F, S2E). This observation was further corroborated with another set of HSC markers such as CD34 and Flk2 [18, 19], which displayed a dramatic increase in the cell number of LT-HSCs (long-term HSCs), ST-HSCs (short-term HSCs) and MPPs (multipotential progenitors) (Figure S2F-G). Additionally, we observed a decreased expression level of Flk2 and an increase in CD150 and CD48 in *Setd5*-deficient LSK^+^ cells (Figure S2H-J).

The increased numbers of HSCs prompted us to further analyze the cell cycle and apoptosis of HSCs. Ki67 staining revealed that significantly higher portions of SLAM-HSC, LSK^+^ and LK cells underwent active cycling in *Setd5*^*CKO*^ than *Setd5*^*fl/fl*^ mice (Fig. 1G-I, S2K). Moreover, increased BrdU incorporations in *Setd5*^*CKO*^ LSK^+^ cells and LT-HSCs were observed (LSK^+^ cells: 42% BrdU^+^ of *Setd5*^*CKO*^ versus 14% of controls, and LT-HSCs: 14% of *Setd5*^*CKO*^ versus 5% of controls) (Figure S2L-M), as seen in LK cells. The apoptotic status shows no differences in LT-HSC and LSK^+^ cells between the two groups (Fig. 1J, K). Together, these observations indicate that deletion of *Setd5* increases the number of immune-phenotypical-defined HSCs via promoting cells into S phase.

### *Setd5* deletion by *Vav-Cre* impairs repopulation capacity of HSCs

**Fig. 2:**
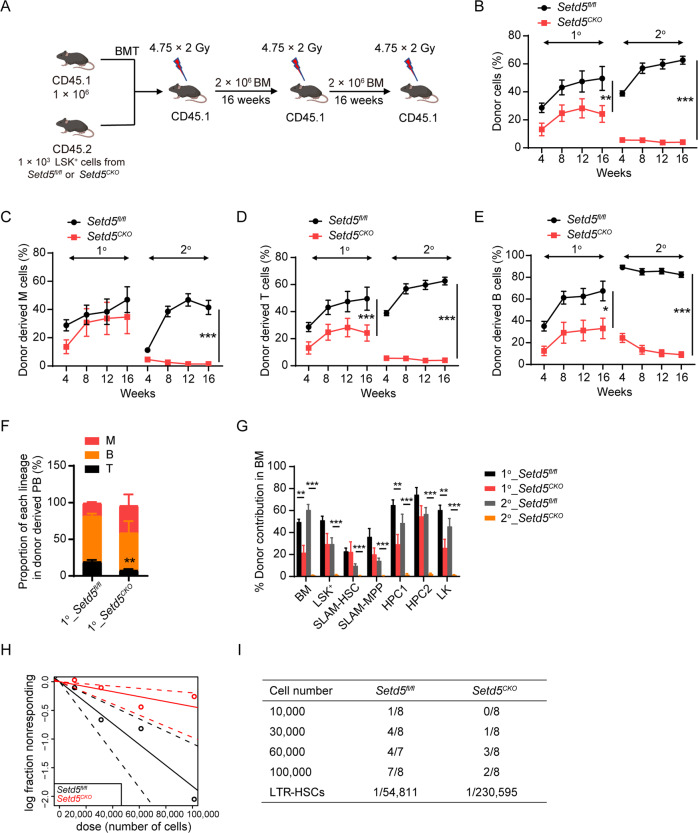
Setd5 deletion by Vav-Cre impairs repopulation capacity of HSCs. **A**–**E** Serial competitive repopulation assays with *Setd5*^*fl/fl*^ and *Setd5*^*CKO*^ LSK^+^ cells. Strategy for serial competitive repopulation assays (**A**), BMT: BM transplantation. Quantification of donor-derived (CD45.2) cells (**B**), donor-derived myeloid (M: CD11b^+^) cells (**C**), donor-derived T (CD3^+^) cells (**D**) and B (B220^+^) cells (**E**) in PB at indicated time points in primary (*n* = 9) and secondary (*n* = 10) recipients. **F** Proportion of each lineage in donor derived PB cells; *n* = 5. **G** Donor contribution of indicated cell populations in BM cells of primary and secondary recipient mice at indicated times; *n* = 5. **H** Poisson statistical analysis from the limiting dilution assay. LTR-HSCs: long-term repopulating (LTR)-HSC. Symbols represent the percentage of negative mice for each dose of cells. Solid lines indicate the best-fit linear model for each dosage. Dotted lines represent 95% confidence intervals. **I** Frequencies of functional HSCs were calculated according to Poisson statistics. Data are represented as Mean ± SEM; * *P* < 0.05, ** *P* < 0.01, *** *P* < 0.001.

The exit of LT-HSCs from the G0 stage is often associated with HSC exhaustion [12, 20, 21]. In considering this, we further performed competitive serial transplantation experiments by transplanting 1000 sorted-LSK^+^ cells from *Setd5*^*CKO*^ or *Setd5*^*fl/fl*^ BM (CD45.2) together with competitor BM cells (CD45.1) into lethally irradiated recipient mice (CD45.1) (Fig. 2A). *Setd5*^*CKO*^ derived cells showed a progressive decrease in repopulation capacity than that of *Setd5*^*fl/fl*^ in primary and secondary transplantation (Fig. 2B-E). Moreover, *Setd5*-deficient cells showed an impaired lineage differentiation ability in PB in primary recipient mice (Fig. 2F). Significantly, although *Setd5*^*CKO*^ derived mature cells represented only less than 20% of the total bone marrow cells, *Setd5*^*CKO*^ derived LSK^+^s could effectively reconstitute the HSC compartments (LSK^+^s, SLAM-HSCs, SLAM-MPPs) in the primary recipients but lost the self-renewal ability in secondary transplantation when compared with that of *Setd5*^*fl/fl*^ derived LSK^+^s (Fig. 2G). The tertiary transplantation also showed an even lower engraftment in *Setd5*^*CKO*^ recipient PB cells (Figure S3A-D).

Additionally, we performed the whole BM competitive transplantation assay which showed a similar result with LSK^+^ competitive transplantation assay (Figure S3E-J), with the chimerism of LSK^+^s, LT-HSCs and MPPs significantly decreased in *Setd5*^*CKO*^ recipients (Figure S3K). Homing assay showed that *Setd5*^*CKO*^ BM cells were able to migrate to the BM of recipients as efficiently as the control cells (Figure S3L). In summary, these results indicate that under transplantation pressure, immune-phenotypical-defined *Setd5*^*CKO*^ HSCs may have an intrinsic defect to differentiate into committed progenitors and subsequently mature blood cells.

Since HSC subtypes defined by immunophenotype may not accurately represent functional HSCs, we further assessed the absolute number of functional HSCs with limiting dilution assay and observed about 4-fold decrease of HSC number in *Setd5*^*CKO*^ mice (Fig. 2H, I). Thus, *Vav-Cre* mediated *Setd5* deletion significantly impaired repopulation capacity of HSCs and decreased number of functional HSCs.

### Setd5 regulates HSC pool in adult hematopoietic cells and plays an intrinsic role in HSCs

**Fig. 3:**
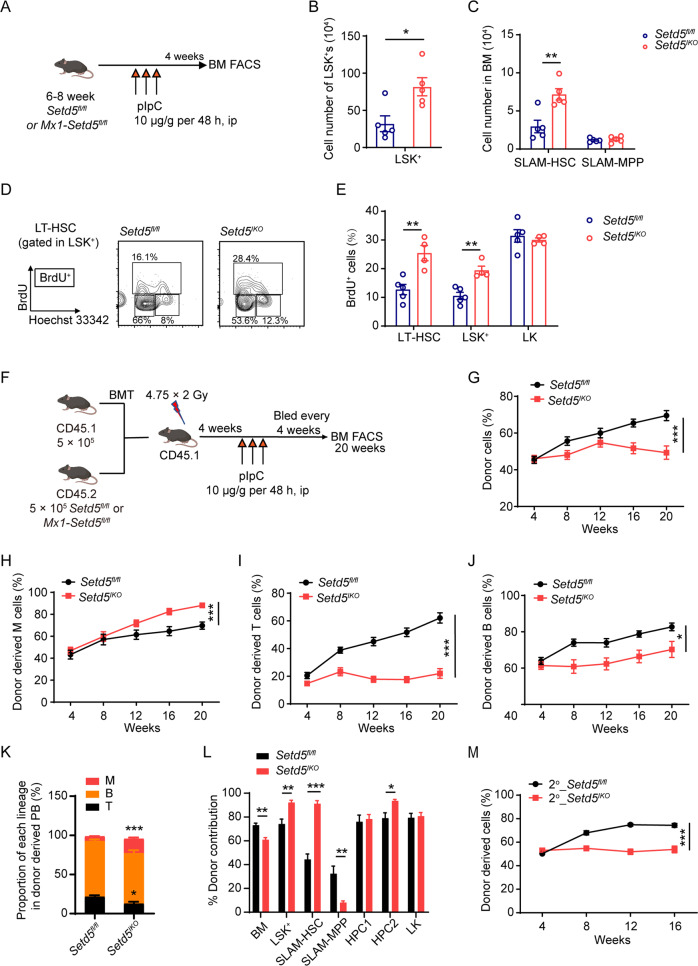
Setd5 regulates HSC pool in adult hematopoietic cells and plays an intrinsic role in HSCs. **A** Schematic diagram of *Mx1*-*Cre* mediated *Setd5* knockout in adult hematopoiesis. **B**, **C** The absolute number of LSK^+^s and SLAM-HSCs in *Setd5*^*fl/fl*^ and *Setd5*^*IKO*^ mice; n = 5. **D**, **E** BrdU incorporation of LSK^+^ and HSC populations and representative flow cytometry histogram of LT-HSC populations of BrdU incorporation 24 h after injection; *n* = 4. **F** Schematic diagram for transplantation assay with *Setd5*^*fl/fl*^ and *Setd5*^*IKO*^ BM cells. **G**–**J** Quantification of donor-derived (CD45.2) cells in the PB of recipient animals at indicated time points; *n* = 9. **K** Proportion of each lineage in donor derived PB cells; *n* = 5. **L** Donor contribution of indicated cell populations in BM cells of recipient mice 20 weeks after transplantation; *n* = 5. **M** Percentage of donor-derived cells in the PB of *Setd5*^*fl/fl*^ and *Setd5*^*IKO*^ secondary recipients at the indicated time points; *n* = 9. Mean ± SEM; * *P* < 0.05, ** *P* < 0.01, *** *P* < 0.001.

Since *Vav-Cre* mediated *Setd5* deletion occurred at E11.5 [22], and the defect of *Setd5*^*CKO*^ HSCs may be due to a long-term accumulated consequence from embryo stage, we generated *Mx1-Setd5*^*fl/fl*^ mice to determine the role of *Setd5* in adult hematopoiesis and confirmed efficient deletion of *Setd5* (*Mx1-Setd5*^*fl/fl*^ with pIpC treatment was referred to as *Setd5*^*IKO*^) (Fig. 3A, S4A-B). Similar to *Setd5*^*CKO*^ mice, we found an increase in the cell number of LSK^+^s and SLAM-HSCs in *Setd5*^*IKO*^ mice, so as the LT-HSCs, ST-HSCs and MPPs (Fig. 3B, C, S4C). BrdU incorporation assay also revealed that higher percentages of *Setd5*^*IKO*^ HSCs and LSK^+^s were in S-phase than those of the controls (Fig. 3D, E), while the cell number of HPCs remained similar in two groups (Figure S4D). These experiments demonstrated that *Setd5* is required for the maintenance of the quiescent state of HSCs in adult hematopoiesis.

To rule out a possible contribution of non-hematopoietic excision of *Setd5* by *Mx1-Cre* to the phenotypes of HSCs, non-competitive BM transplantation (nCBMT) was performed before pIpC injection (Figure S4E). Sixteen weeks after depletion of *Setd5*, the absolute cell numbers of LSK^+^, SLAM-HSC, LK, and MEP were significantly increased in *Setd5*^*IKO*^ recipients (Figure S4F-H). BrdU incorporations of donor derived LSK^+^ cells were also increased in *Setd5*^*IKO*^ chimeric mice (Figure S4I). These results showed that *Setd5* regulates HSC maintenance in a cell-autonomous manner.

To ascertain whether acute deletion of *Setd5* in adult mice by pIpC injection would also affect HSC repopulation ability, we performed competitive transplantation with BM cells from *Setd5*^*fl/fl*^ and *Mx1*-*Setd5*^*fl/fl*^ before pIpC treatment (Fig. 3F). pIpC was injected after transplantation and *Setd5* was efficiently deleted in primary and secondary *Setd5*^*IKO*^ recipients (Figure S4J). A progressive decrease in PB chimerism was observed in primary and secondary transplantation. Lymphoid lineage reconstitution was impaired while myeloid lineage reconstitution was increased in *Setd5*^*IKO*^ recipients than those of *Setd5*^*fl/fl*^ in serial transplantations (Figs. 3G-J and 3M, S4K-M), implying a differentiation bias towards myeloid lineage at the expense of lymphoid lineage of *Setd5*^*IKO*^ HSCs (Fig. 3K, S4N). A higher chimerism of LSK^+^s and SLAM-HSCs was detected in *Setd5*^*IKO*^ recipient BM cells (Fig. 3L, S4O). In summary, these results indicated that under transplantation pressure, *Setd5* deletion by *Mx1-Cre* could also affect HSC repopulation ability but enhanced the proliferation of HSCs and promoted myeloid lineage differentiation.

### Transcriptome profiling of *Setd5*-deficient HSCs reveals altered stem cell property and cell cycle signature

**Fig. 4:**
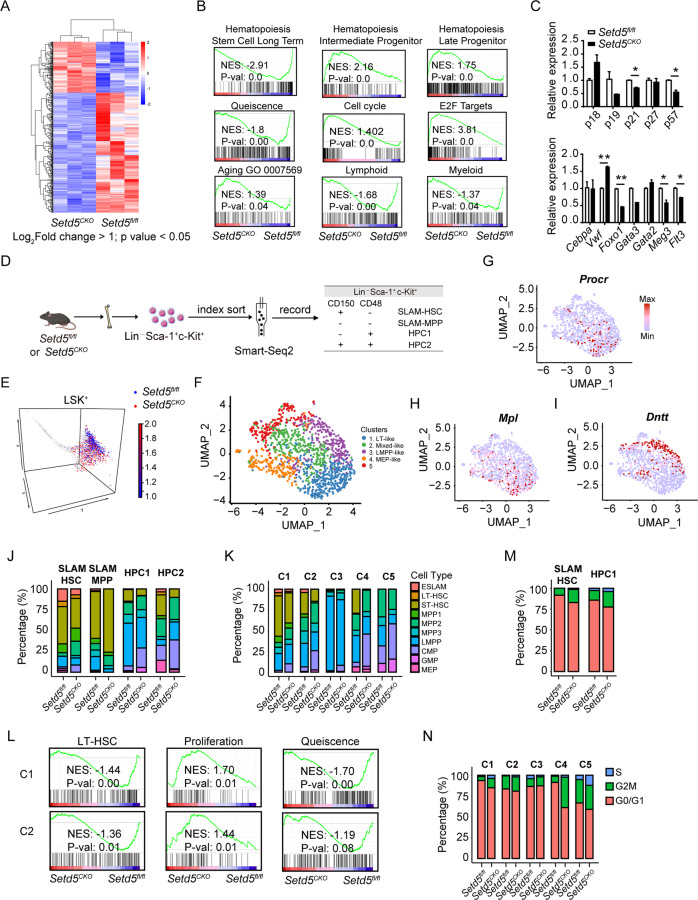
Transcriptome profiling of Setd5-deficient HSCs reveals altered stem cell property and cell cycle signature. **A** Heat map showing differential expression of 443 genes in *Setd5*^*fl/fl*^ and *Setd5*^*CKO*^ SLAM-HSCs, |Log_2_foldchange | > 1, *p* value < 0.05; *n* = 3. **B** GSEA analyses for genes affected in the SLAM-HSCs of *Setd5*^*fl/fl*^ and *Setd5*^*CKO*^ mice. NES, normalized enrichment score. **C** Relative expression levels of cell cycle and multi-potency genes in HSC cells; *n* = 3. mRNA levels were normalized to the expression of 18 s. **D**. Experimental design for single cell sequencing with Smart-seq2, LSK^+^ cells were indexed sorted into 96-well plates containing lysis buffer. **E** LSK^+^ cells were projected onto Nestorowa *et al*. data [30]. **F** UMAP visualizations of single cell transcriptomes of identified clusters using Seurat. **G**–**I** Diffusion maps of all cells were colored according to the expression levels of selected genes. **J**, **K** Histograms showing the compositions of surface markers defined four populations and transcriptome-defined five clusters by ten cell types annotated with Nestorowa *et al*. data. C1-C5 means cluster 1-5. Ten annotated cells including E-SALM (EPCR^+^CD48^–^CD150^+^), LT-HSC (Lin^–^Sca1^+^c^-^Kit^+^CD34^–^Flt3^low^), ST-HSC (Lin^–^Sca1^+^c-Kit^+^CD34^+^Flt3^low^CD48^–^CD150^–^), MPP1 (Lin^–^Sca1^+^c-Kit^+^CD34^+^Flt3^low^CD48^–^CD150^+^), MPP2 (Lin^–^Sca1^+^c-Kit^+^CD34^+^Flt3^low^CD48^+^CD150^+^), MPP3 (Lin^–^Sca1^+^c-Kit^+^CD34^+^Flt3^low^CD48^+^CD150^–^), LMPP (Lin^–^Sca1^+^c-Kit^+^CD34^+^Flt3^+^), CMP, GMP and MEP. **L** GSEA of LT-HSC, proliferation and quiescence signatures comparing between *Setd5*^*CKO*^ and *Setd5*^*fl/fl*^ in HSC enriched cluster 1 and cluster 2. **M**, **N** Proportion of SLAM-HSCs, HPC1s and five clusters identified with Seurat in each of the cell cycle stages between two groups.

To dissect the molecular mechanism of *Setd5* in regulating HSC function, we performed bulk RNA-seq (RNA sequencing) of SLAM-HSCs derived from *Setd5*^*CKO*^ and control mice, and 443 differentially expressed genes (DEGs) were found (Fig. 4A, Table S2). These DEGs were significantly enriched in cell cycle and Pol II transcription activity (Figure S5A). Additional Gene Set Enrichment Analysis (GSEA) revealed a significantly lower expression of LT-HSC signature genes and a decreased quiescence state, accompanied with a higher expression of intermediate and late progenitor signature genes, as well as an enrichment of cell cycle and E2F targets in *Setd5*^*CKO*^ SLAM-HSCs (Fig. 4B). *Setd5*^*CKO*^ SLAM-HSCs also exhibited an increased bias towards age-associated patterns accompanied with a decreased lymphoid/myeloid signature (Fig. 4B). Among these DEGs, lymphoid differentiation and pluripotency maintenance related genes, such as *Meg3*, *Flt3* and *Foxo1* [23–25] were significantly downregulated, so as *p21* and *p57*. The expression of *Vwf*, which was involved in specifying myeloid fate [26, 27], was increased in *Setd5*^*CKO*^ HSCs (Fig. 4C). We also performed the transcriptome analysis of SLAM-HSCs in *Setd5*^*fl/fl*^ and *Setd5*^*IKO*^. 145 upregulated and 185 downregulated genes were found (Figure S5B, Table S3). These DEGs were also enriched in DNA replication and cell cycle (Figure S5C). GSEA also exhibited a decreased LT-HSC and quiescence signature, accompanied with an age-associated bias in *Setd5*^*IKO*^ SLAM-HSCs (Figure S5D). Similar expression patterns of *Vwf*, *Foxo1*, *Gata3*, *p21* and *p57* were also observed in *Setd5*^*IKO*^ SLAM-HSCs (Figure S5E). Taken together, the transcriptional alterations triggered by *Setd5*-deficiency suggested that the absence of *Setd5* induced SLAM-HSCs to exit from quiescence phase and led to the exhaustion of functional HSCs.

To delineate the transcriptional networks of HSCs at the single-cell resolution, we further performed scRNA-seq (single-cell RNA sequencing) of LSK^+^ cells in *Setd5*^*CKO*^ and *Setd5*^*fl/fl*^ mice by using Smart-seq2 [28, 29] (Fig. 4D, S5F). We isolated single LSK^+^ cells stained with CD150 and CD48 and recorded the index-sorting data for each single cell. In total, 1380 cells were processed for scRNA-seq library construction and sequencing, with 1262 cells passed the quality control (see “Supplemental information”). LSK^+^ cells were projected onto Nestorowa *et al*. data [30] and matched perfectly with cell types as defined in the paper (Fig. 4E), indicating that our scRNA-seq data is of high quality. Moreover, LSK^+^s from *Setd5*^*CKO*^ mice were largely deviated from the core territory toward more differentiated progenitor state in comparison with their counterparts in *Setd5*^*fl/fl*^ mice, suggesting a shift from HSC to HPC at the transcriptional level with *Setd5* deletion.

We further grouped cells into 5 clusters based on their transcriptomes with Seurat packages (Fig. 4F, S5G), hereafter termed as LT-like (cluster 1, long-term stem cell-like), Mixed-like (cluster 2, mixed stem cell-like), LMPP-like (cluster 3, lymphoid-primed multipotent progenitor-like) and MEP-like cluster (cluster 4). *Nr4a1*, *Mpl*, *Egr1*, *Dntt*, *Flt3, Icam4* and *Vwf* were represented as specifically and highly expressed genes in LT-like, Mixed-like, LMPP-like and MEP-like cluster, respectively. The expression levels of individual genes were plotted in the diffusion map (Fig. 4G-I, S5H-J, Table S4). We found a reduced fraction of LT-like and LMPP-like clusters in *Setd5*^*CKO*^ LSK^+^s (Figure S5K). Moreover, by projecting surface markers defined LSK^+^s to cell types defined in Nestorowa *et al*. paper including LT-HSCs, ESLAMs, ST-HSCs, MPP1s, MPP2s, MPP3s and LMPPs, we found that the fractions of LT-HSCs and ESLAMs were significantly lower in *Setd5*^*CKO*^ SLAM-HSCs than that of the controls (Fig. 4J). We further projected transcriptome-defined 5 clusters to cell types defined by Nestorowa *et al*., and found that LT-HSCs and ESLAMs were enriched in cluster 1, while LMPPs were enriched in cluster 3 (Figure S5L). We also observed a significant reduced fraction of ESLAMs, LT-HSCs and ST-HSCs in cluster 1 and cluster 2 accompanied with decreased LMPPs in cluster 3 due to *Setd5* deletion (Fig. 4K), and the decreased LT-HSC signature in *Setd5*^*CKO*^ was also recapitulated by performing GSEA (Fig. 4L). Cluster 3 exhibited a decreased bias towards LMPPs whereas cluster 4 showed bias in erythroid differentiation in *Setd5*^*CKO*^ group, accompanied with a decreased myeloid signature in cluster 5 (Figure S5M). These data indicated that both transcriptome- and immunophenotype-defined LT-HSCs lost the long-term stem cell signatures and differentiated toward multipotent progenitors with impaired lymphoid commitment due to *Setd5* deficiency.

In addition, the proliferation signature of cluster 1 and 2 was increased, accompanied with a decreased quiescence signature in *Setd5*^*CKO*^ group as revealed by GSEA (Fig. 4L). To further characterize the cell cycle changes, we used a recently reported predictor for allocating individual cells to G0/G1, S and G2/M cell cycle categories based on single-cell transcriptomes [31]. We found that the percentages of S and G2/M cell cycle signatures were elevated in *Setd5*^*CKO*^ SLAM-HSC and HPC1 cells, as well as in Cluster 1, 2, 4 and Cluster 5 (Fig. 4M-N). Collectively, these data suggest a decreased quiescence state and enhanced proliferation with *Setd5* deficiency, that might contribute to the functional lineage biases in *Setd5*^*CKO*^ HSCs at the single cell level.

### SETD5 regulates the expression of E2F targets associated with HCF-1

**Fig. 5:**
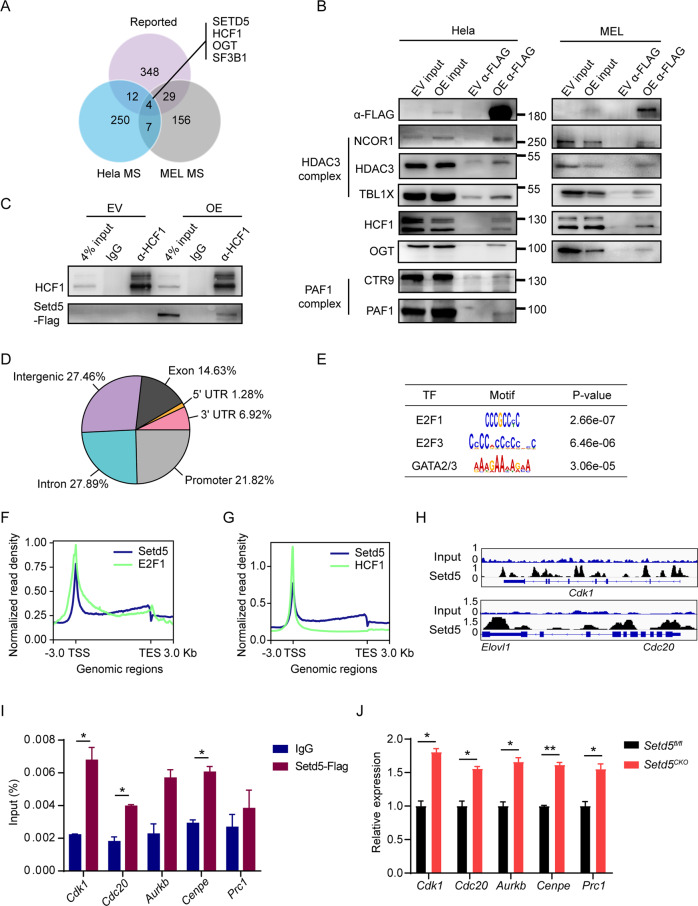
SETD5 regulates the expression of E2F targets associated with HCF-1. **A** Venn diagrams showing the overlap between Hela/MEL MS-identified proteins and reported SETD5 interactors. **B** Anti-FLAG immunoprecipitation of lysates from Hela and MEL cells transfected with SETD5-FLAG (OE) and empty FLAG vector (EV). Input: cell extract before immunoprecipitation, FLAG-IP: immunoprecipitation elute with 2×loading buffer. **C** Reciprocal immunoprecipitation with antibodies against HCF-1 in MEL cells transfected with SETD5-FLAG and empty FLAG vector. **D** Pie chart showing the distribution of ChIP-seq peaks for SETD5 with respect to promoter (define as a 3.0-kb proximal region centered on the gene start sites, including TSS), 5’UTR (5’-untranslated region), 3’UTR (3’-untranslated region), exon, intron and intergenic regions. **E** MEME motif analysis of SETD5 peaks, TF: transcription factor. **F**–**G** Density plots of SETD5 (blue) and E2F1/HCF-1 (green) normalized ChIP-seq signals, TES: Transcription End Site. **H** IGV tracks of target genes of SETD5-FLAG, and input DNA in MEL cells. The coverage data are represented as normalized reads of bins per million (BPM). **I** ChIP-qPCR of the binding of SETD5-FLAG to the promoters of indicated genes, *Prc1* is a negative control which was not found in FLAG-SETD5’s ChIP-seq data. **J** Relative expression levels of target genes in control and *Setd5*^*CKO*^ LSK^+^ cells measured by real-time PCR; n = 3. mRNA levels were normalized to the expression of 18 s. Mean ± SEM; * *P* < 0.05, ** *P* < 0.01, *** *P* < 0.001.

Although SETD5 has a SET domain, its enzyme activity was and may still be in question [6, 7, 32]. By immunoblot analysis, no obvious changes in majority of histone methylation were observed (Figure S6A), suggesting that SETD5 might lack histone methyltransferase activity and exert its function via interaction with other proteins. To explore the potential partners of SETD5, we performed co-immunoprecipitation (Co-IP) assay coupled with quantitative mass spectrometry (MS) using SETD5-FLAG stably expressed Hela or MEL (a murine erythroleukemia cell) cell lines. Overlapping of our Hela and MEL SETD5-IP/MS data with previously reported SETD5 associated proteins [5–7], revealed four proteins including SETD5, HCF-1 (host cell factor C1), OGT (O-linked N-acetylglucosamine (GlcNAc) transferase) and SF3B1 (splicing factor 3b subunit 1) (Fig. 5A). Additional components of HDAC3 complex (including HDAC3, NCOR1 and TBL1X), PAF1 (RNA Pol II–associated factor 1) complex (including PAF1 and CTR9) and OGT that have been previously identified [5–7] were also found in our IP/MS experiments (Fig. 5A, B, Table S5-6). We further confirmed that HCF-1 could be immunoprecipitated by SETD5 both in MEL and Hela cells in Co-IP assays and their interaction was further verified with reciprocal co-immunoprecipitation with HCF-1 antibody in MEL cells (Fig. 5B, C).

HCF-1 was reported to interact with both repressive and activating E2Fs, and to regulate the expression of its downstream targets including cell cycle entry [33–35]. The function of HCF-1 in cell cycle regulation prompted us to further examine the potential roles of SETD5 in regulating E2F targets via its association with HCF-1. To identify the potential targets of SETD5 unbiasedly in the blood cells, we performed chromatin immunoprecipitation followed by next-generation sequencing (ChIP-seq) using SETD5-FLAG stably expressed MEL cells. We found that the genomic distribution of SETD5 was highly enriched on promoter region (defined as a 3.0-kb region centered by the transcription start sites (TSS)) and gene bodies (intron and exon) (Fig. 5D). Motif enrichment analysis of SETD5 peaks revealed an enrichment of E2F1, E2F3, and GATA2/3 (Fig. 5E). Further analysis revealed a similar distribution along genomic regions between SETD5 and E2F1/4 as well as HCF-1 (Fig. 5F-G, S6B), suggesting that SETD5 binds the E2F targets with HCF-1. Among E2F1 targets, 66.5% (4794/7204) were enriched with SETD5 bindings and 34.8% (4794/13774) of SETD5 targets were E2F1 targets. However, only 3.88% (186/4794) SETD5/E2F targets showed differential expression (Figure S6C). These 186 SETD5/E2F targeted DEGs were enriched with cell-cycle process and cell division (Figure S6D). The binding of SETD5 at the promoter region of several E2F target genes, including *Cdk1*, *Cdc20*, *Aurkb,* and *Cenpe*, were observed (Fig. 5H, S6E) and further confirmed with ChIP-qPCR (Fig. 5I). Moreover, we found that the expression of these genes was significantly upregulated in *Setd5*^*CKO*^ LSK^+^ cells (Fig. 5J). Taken together, our results demonstrated that SETD5 could be localized to E2F-responsive promoters in association with HCF-1 to regulate gene transcription.

Considering that the interaction between SETD5 and HDAC3/NCOR complex was observed in previous studies [5, 6, 36] as well as ours, and higher H3 pan-Kac (pan-acetylation of H3 lysine) and H3K9ac were observed in *Setd5*^*−/−*^ mouse embryonic stem cells (mESC) [5], we further examined the histone acetylation levels in *Setd5*^*−/−*^ hematopoietic stem/progenitor cells. Immunoblot analysis of *Setd5*^*fl/fl*^ control and *Setd5*^*CKO*^ c-Kit^+^ cells showed similar histone acetylation levels of H3K9, H3K27, H3K14, H4K5, H4K8, and H4K12 in both groups (Figure S6F), suggesting that the disrupted interaction of SETD5 and HDAC3 in hematopoietic cells may not affect histone acetylation levels.

### SETD5 regulates the promoter-proximal paused Pol II release on E2F targets

**Fig. 6:**
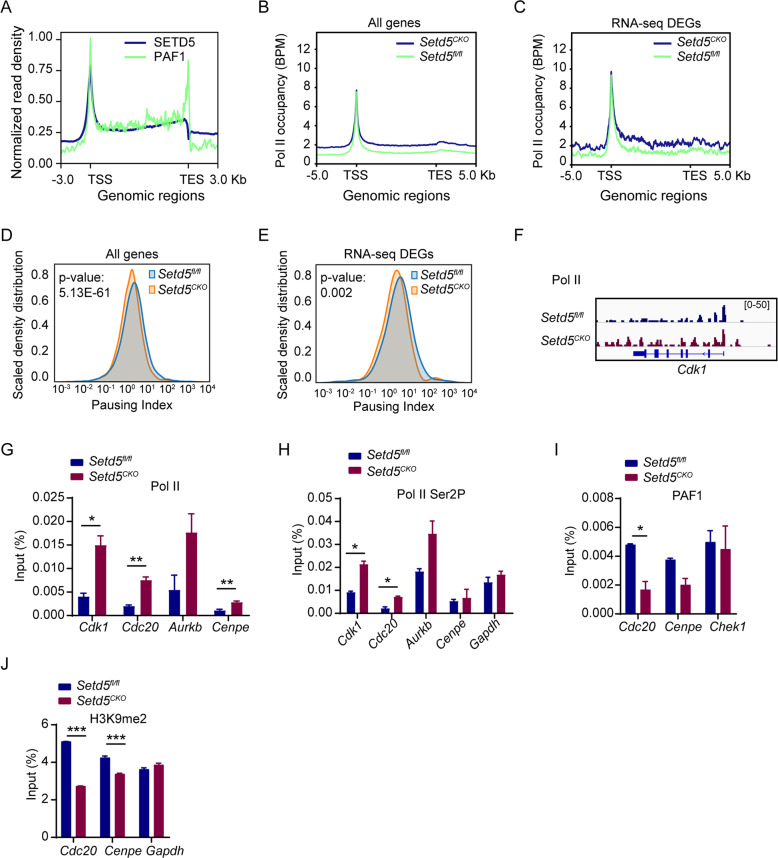
SETD5 regulates the promoter-proximal paused Pol II release on E2F targets. **A** Density plots of SETD5 (blue) and PAF1 (green) normalized ChIP-seq signals. **B**, **C** Occupancy of total Pol II on all genes and DEGs (Fold change>1.5 and p adj value<0.05), ChIP normalization was implemented by integrating Spike-in Chromatin. **D**, **E** Density plot of all genes and DEGs with reduced average Pol II pausing index in *Setd5*^*CKO*^. **F** IGV tracks comparing occupancy of Pol II within *Cdk1* locus. **G**, **H** ChIP-qPCR of Pol II and Pol II Ser2P at the promoters of indicated genes in c-Kit^+^ cells from control or *Setd5*^*CKO*^ mice; *n* = 2. **I** ChIP-qPCR analysis of PAF1 at the promoters of indicated genes in c-Kit^+^ cells; *n* = 2. **J** The H3K9 dimethylation levels at *Cdc20* and *Cenpe* promoters; *Gapdh* as an internal control; *n* = 2. Mean ± SEM; *P < 0.05, **P < 0.01, *** *P* < 0.001.

We further investigated the mechanism of SETD5 in regulating the expression of E2F targets since HCF-1 was reported to recruit MLL and Set-1 H3K4 HMT complexes to E2F responsive promoters to regulate gene expression [35, 37]. However, no obvious change among the genomic distribution of H3K4me3 was observed due to *Setd5* deficiency (Figure S6G). Interestingly, HCF-1 was recently found to associate with multiple transcription initiation and elongation complexes to regulate Pol II pausing [38]. Besides, PAF1, a critical regulator of Pol II pausing [39, 40], was immunoprecipitated with SETD5 (Fig. 5B), and showed a similar distribution pattern with SETD5 (Fig. 6A). We thus postulated that SETD5 modulates the release of promoter-proximal paused Pol II. By analyzing the genome-wide distribution of Pol II in control and *Setd5*^*CKO*^ c-Kit^+^ cells by ChIP-Seq, we found an increase in Pol II occupancy on all genes and DEGs upon SETD5 deficiency (Fig. 6B, C and S6H-I). We further calculated pausing index (the ratio between promoter-proximal and gene body Pol II density) to measure the release of Pol II from promoters. Deficiency of SETD5 led to a significant decrease of pausing index on average (Fig. 6D), as well as on the DEGs (Fig. 6E), which represented a transcription elongation state. Pausing index was decreased in 5622 genes (59.5%) and increased in 3828 genes (40.5%) (Fig. S6J). We observed significantly increased Pol II occupancy at *Cdk1* locus (Fig. 6F). Using ChIP-qPCR, we confirmed the increased Pol II and Pol II Ser2P (phosphorylated Ser2) occupancy at *Cdk1*, *Cdc20*, *Aurkb* and *Cenpe* in *Setd5*^*CKO*^ cells in comparison with the controls (Fig. 6G, H). These data suggested that SETD5 deficiency may promote the release of paused Pol II into productive elongation. Although the total protein level of PAF1 was not significantly changed (Fig. S6K), decreased occupancies of PAF1 at *Cdc20* and *Cenpe* in *Setd5*^*CKO*^ c-Kit^+^ cells were observed (Fig. 6I), suggesting that SETD5 remodeled Pol II pausing and elongation state on E2F targets to regulate their expression, most likely via its interaction with the PAF1 complex.

In consistent with a previously reported role of SETD5 to affect H3K9me2 levels via association with HDACs-G9a co-suppressor complex [7], we also observed a mild decrease of H3K9me2 occupancy at E2F targets (Fig. 6J). Recently, a robust correlation between histone acetylation and transcription elongation had been reported, and HDAC inhibition was shown to stimulate the release of promoter-proximal paused Pol II [41, 42]. The interaction between SETD5 and HDAC3/NCOR complex (Fig. 5B) prompted us to further examine whether the loss of SETD5 would alter H3 pan-Kac, H3K9ac, and H3K27ac around the promoter and 5’UTR of *Cdk1*, *Cdc20*, *Aurkb,* and *Cenpe*. However, no significant differences were found (Fig. S7A-Q), suggesting that the effect of SETD5 on the regulation of Pol II promoter pausing is probably independent of SETD5-HDAC3/NCOR complexes in normal hematopoiesis. We also examined the genomic distribution of H3K36me3 and found no change between two groups (Fig. S7R).

**Fig. 7:**
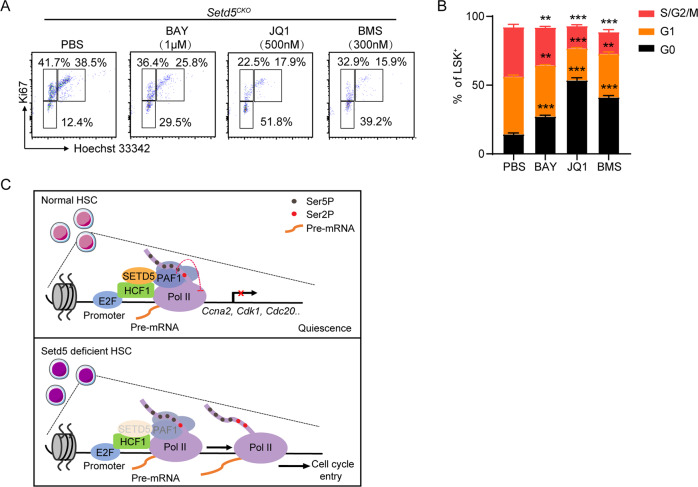
Setd5CKO deficiencies could be partially rescued by super elongation complex-related inhibitors. **A** c-Kit^+^ BM cells from *Setd5*^*fl/fl*^ and *Setd5*^*CKO*^ mice were treated with BAY-1143572 1 μM, JQ1 500 μM, and BMS-387032 300 nM for 24 h before cell cycle analysis. **B** The frequencies of G0, G1, S/G2/M LSK^+^s in drug-treated c-Kit^+^ cells for 24 h from *Setd5*^*fl/fl*^ and *Setd5*^*CKO*^ mice were shown; *n* = 4. **C** A model for the role of SETD5 in modulating hematopoietic stem cell homeostasis. In normal HSCs, SETD5 occupies at E2F-responsive promoters in association with HCF-1, maintains a paused Pol II state with PAF1 complex, leading to transcriptional silencing of E2F targets and a quiescent state of HSCs. Once *Setd5* is depleted, PAF1 occupancy is weakened and triggers Pol II elongation to active transcription of E2F target genes and promote G1 to S phase transition.

To further determine whether increased cell-cycle transition was mainly caused by the increased Pol II elongation at E2F targets, we employed two inhibitors BAY 1143572 (p-TEFb/CDK9 inhibitor) and JQ1 (Brd4 inhibitor) to inhibit the transition of paused Pol II to elongation and analyzed the cell cycle of LSK^+^ cells, with CDK inhibitor BMS-387032 as a positive control. The c-Kit^+^ cells from *Setd5*^*CKO*^ were treated in an in vitro culture system with individual inhibitors for 24 h. Significantly decreased S phases were observed in LSK^+^ cells in all drug-treated groups when compared with PBS treated group (Fig. 7A, B). Taken together, these data indicated that increased transition of paused Pol II to elongation mediated by *Setd5* depletion could be a main cause for the exit of quiescence stage of *Setd5*^*CKO*^ HSC cells.

## Discussion

In this study, we investigated the roles of *Setd5* in HSCs during hematopoiesis. We found that deletion of *Setd5* led to an enhanced proliferation and accumulation of HSCs both in *Vav-Cre* and *Mx1-Cre*;*Setd5*^*fl/fl*^ (abbreviated as *Setd5*^*CKO*^ and *Setd5*^*IKO*^ respectively) murine models. However, this was accompanied by a strikingly decreased number of functional HSCs and a reduced competitive reconstitution ability with long-term depletion of *Setd5* in *Setd5*^*CKO*^ mice. When *Setd5* was deleted in *Setd5*^*CKO*^, more HSCs exited from quiescence and entered into G1 phase, and consequently, cell cycle perturbed *Setd5*^*CKO*^ HSCs displayed impaired self-renewal and multi-lineage differentiation abilities. Interestingly, while more *Setd5*^*IKO*^ HSCs were also in proliferative stages, these cells exhibited enhanced proliferation and myelopoiesis in serial transplantations. These different results probably are a differential time and developmental stage effect at which *Setd5* is depleted in *Vav-Cre* or *Mx1-Cre* mediated *Setd5* excision.

Moreover, both bulk and single-cell RNA-seq revealed that transcriptome- and immunophenotype-defined LT-HSCs showed reduced long-term stem cell and quiescence signatures upon *Setd5* deletion, accompanied by impaired lymphoid commitment and more cell cycle entrance. These results exemplified a key role of *Setd5* in the balance between proliferation and differentiation of HSCs. Mechanistically, although HCF-1 was on a list of potential SETD5 associated proteins identified with previous IP-MS assay [5], the biological function of the interaction between HCF-1 and SETD5 was not explored. Here, we verified the interaction between HCF-1 and SETD5 and found the enrichment of SETD5 at the E2F targets. More recently, HCF-1 was reported to promote transcription elongation of viral Immediate Early genes via the interaction with transcriptional elongation factors including PAF1 in response to herpes virus infection [38]. Our observation suggests that the loss of Pol II pausing of *Setd5* deficient HSCs may be caused by the disrupted association of HCF-1 and transcriptional elongation factors including but not limited to PAF1. Previous study reported the interaction between PAF1 and SETD5 and found that depletion of *Setd5* could promote Pol II pausing in mESCs [6]. PAF1 has been identified as a regulator of release of promoter-proximal paused RNA Pol II or promoter-proximal pausing by RNA Pol II [39, 40]. Our results reveal that SETD5 restricts Pol II release from pausing to elongation associated with PAF1 at cell cycle related genes to maintain HSC quiescence. These results suggest that the role of *Setd5* in the regulation of paused Pol II release is tissue specific.

Although higher bulk H3 pan-Kac and H3K9ac and increased H3K9ac levels at target genes were observed in mESCs and murine PDAC (pancreatic ductal adenocarcinoma) cells upon *Setd5* deficiency respectively [5, 36]. Deliu E et al. reported that higher histone acetylation observed in *Setd5*^*+/−*^ hippocampal samples and ESCs was independent of HDAC3 activity [6]. In our study, deletion of *Setd5* in hematopoiesis has no effect on bulk levels of histone acetylation and site-specific histone acetylation, suggesting that the effect of SETD5 on histone acetylation may be tissue or cell type specific.

Although SETD5 was reported to have an intrinsic methyltransferase activity on H3K36 [32], several other groups had shown that SETD5 lacks histone lysine methyltransferase activity [5, 6, 36]. In our study, no obvious differences were observed of H3K36me3 on both protein level and ChIP-seq signal upon *SETD5* deletion, strongly indicating that SETD5 may indeed lack methyltransferase activity on H3K36. The genomic distribution of SETD5 in our study was slightly different with the ChIP-seq data by Sessa *et al*. Neuron 2019 [32], which may be due to different cell types and antibodies used in the experiments.

Interestingly, SETD5 mutations were reported to cause congenital abnormalities, and multiple congenital anomalies were also observed in two boys with mutation in HCF-1 [43]. PAF1 complex was also reported to has mutations associated with autism [44, 45], suggesting a model of potential interplay among HCF-1, PAF1 and SETD5 proposed in our study may be applicable in the onset/development of certain mental diseases.

In summary, our study provided a new insight into the molecular mechanism of *Setd5* in regulating HSC quiescence by Pol II pausing-mediated differential expression of cell cycle genes via HCF-1-SETD5-PAF1-Pol II axis (Fig. 7C). We also revealed that regulation of Pol II pausing and release plays an essential role in HSC maintenance.

## Supplementary information

Supplemental Information

Supplemental Table 1

Supplemental Table 2

Supplemental Table 3

Supplemental Table 4

Supplemental Table 5

Supplemental Table 6

## Data Availability

All sequence data generated in this study have been deposited in the Genome Sequence Archive [46] in National Genomics Data Center [47], China National Center for Bioinformation/Beijing Institute of Genomics, Chinese Academy of Sciences, under accession number CRA003816 that is publicly accessible at https://bigd.big.ac.cn/gsa.
