# Supplementary material for: SETD5 modulates homeostasis of hematopoietic stem cells by mediating RNA Polymerase II pausing in cooperation with HCF-1
Source: Leukemia. 2021 Dec 1;36(4):1111–22. doi: 10.1038/s41375-021-01481-1 (PMC8979820; doi:10.1038/s41375-021-01481-1)
Supplement: Supplementary file 1 — Supplemental Information [file 41375_2021_1481_MOESM1_ESM.docx]

**Supplemental Information**

**SETD5 modulates homeostasis of hematopoietic stem cells by mediating RNA Polymerase II pausing in cooperation with HCF-1**

Mengke Li^1^, Chen Qiu^1^, Yujie Bian^1^, Deyang Shi^1^, Bichen Wang^1^, Qiuyi Ma^1^, Xiaomin Wang^1^, Jun Shi^1^, Lianfeng Zhang^2^, Yuanwu Ma^2^, Ping Zhu^1^, Tao Cheng^1^, Yajing Chu^1,*^, Weiping Yuan^1,*^

**Contents:**

Supplemental Materials and Methods;

Supplemental Figure 1;

Supplemental Figure 2;

Supplemental Figure 3;

Supplemental Figure 4;

Supplemental Figure 5;

Supplemental Figure 6;

Supplemental Figure 7

Supplemental Table 1-6 are provided and submitted as individual file separately;

**Supplemental Materials and Methods**

**Animals**

All transgenic and conditional knockout mice were on C57BL/6-Ly5.2 (Ly5.2, CD45.2) genetic background. Both male and female mice at 8-12 weeks old were used for experiments, with age- and gender-matched littermates as control. B6.SJL-Ly5.1 (Ly5.1, CD45.1) recipient mice at 8-10 weeks old were used for hematopoietic transplantation assays. *Setd5^fl/fl^* mice were crossed with either *Vav-Cre* or *Mx1-Cre* mice to produce *Vav-Cre*;*Setd5^fl/fl^* or *Mx1-Cre*;*Setd5^fl/fl^* mice respectively. *Vav-Cre*;*Setd5^fl/fl^* mice were born and produced offspring normally. Mice tail DNA was used for PCR genotyping, efficient excision of floxed exons was confirmed with gDNA PCR of BM cells from mice. All animal research was approved by the Institutional Animal Care and Use Committee of the State Key Laboratory of Experimental Hematology, with an assigned approval number: NSFC2021017-EC-1.

**Generation of stable cell lines**

Mouse cell line MEL cells, human cell line Hela and HEK293T cells were obtained from cell bank of SKLEH (purchased from ATCC and maintained by the cell bank). The cells were tested for mycoplasma contamination monthly using a mycoplasma detection kit (InvivoGen). Cell lines were validated for authentication using the short tandem repeat method (Beijing Microread Genetics Corporation Limited). MEL cells were cultured at 4-6×10^5^ cells/ml in RPMI-1640 GlutaMAX containing 10% FBS, 1% penicillin/streptomycin, and Hela cells were cultured in DMEM containing 10% FBS, 1% penicillin/streptomycin, and then transduced with lentiviruses expressing SETD5-3×FLAG-puroR and control viruses in parallel. 48 hrs after infection, cells were treated with puromycin (InvivoGen) for 48 hrs and remaining cells were >90% eGFP^+^.

**Complete blood counts analysis**

20 μl peripheral blood (PB) from tail vein of mice were collected into 1.5 ml tube containing 120 μl PE (PBS contained 2 mM EDTA) and collected [1]. The blood samples were measured and analyzed by XN-1000 V hematology analyzer (Sysmex).

**Colony forming assay**

3×10^4^ BM cells were seeded in 1 ml Mouse Methylcellulose Complete Media M3434 (Stem Cell Technologies) and cultured at 37^o^C, 5% CO_2_ for 12-14 days. Colonies were counted, and granulocyte-macrophage colonies (GM), granulocyte-erythroid-macrophage-megakaryocyte colonies (GEMM) and burst-forming unit-erythroid colonies (BFU-E) were identified according to a standard protocol.

**Plasmid construction and virus packaging**

pLVX-CMV-3×FLAG-GFP-puroR vector was used. For the overexpression of SETD5, synthesized Setd5-3×Flag DNA was cloned into pLVX-CMV-GFP-puroR plasmid by YouBio company and verified with Sanger Sequencing (BGI tech company). 3×FLAG was added to the C terminal of SETD5. Lentiviruses were produced in HEK293T cells transfected using Polyetherimide (PEI) with viral packaging constructs pMD2.G and pSPAX2. Viral supernatants were harvested at 48 and 72 hours after transfection and filtered through 0.45 μm low protein binding membrane (Millipore).

**Flow cytometry analysis and cell sorting**

Staining and enrichment procedures for flow cytometry have been previously described [2, 3]. Antibodies used were described as above. Lineage markers (Lin cocktail) including CD3, CD4, CD8, B220, Mac1, Gr1 and Ter119. Antibody combinations for hematopoietic stem/progenitor cell analysis were listed in Supplemental Table S1. Cells were sorted on an Aria III flow cytometer and analyzed on LSR II or Canto II instrument (BD Biosciences). For sorting SLAM-HSCs, CD117 positive cells were enriched with CD117 microbeads (Miltenyi Biotec) according to the manufacturer’s instructions. Flow cytometry data were analyzed using FlowJo software (v10.07). For Ki67 staining assay, cells were stained with cell surface marker first, fixed with BD FACS lysing solution, treated with BD IntraSure Kit, stained with anti-Ki67 and resuspended with PBS buffer. Hoechst 33342 were added prior to FACS analysis. For BrdU incorporation assay, mice were given 16 μg/g body weight BrdU (Sigma) by intraperitoneal injection and at the same time, fed with the water containing 1 mg/ml BrdU for 24 hours. For apoptosis staining, cells were stained with HSC markers first, then resuspended in BD Binding Buffer and stained with anti-Annexin V (BD) for 15 min at room temperature

**BM transplantation**

For whole BM transplantation assay, 1×10^6^ BM cells from *Mx1-Cre*;*Setd5^fl/fl^* mice or *Setd5^fl/fl^* were injected intravenously into lethally irradiated (9.5 Gy X-ray) CD45.1 mice. Four weeks after transplantation, 10 μg pIpC were intraperitoneal injected per gram of body weight for three times with 48 h intervals. For competitive reconstitution assays, 5×10^5^ BM cells from *Vav-Cre*;*Setd5^fl/fl^*, *Mx1-Cre*;*Setd5^fl/fl^* (untreated) and their littermate controls along with 5×10^5^ CD45.1 whole BM competitor cells were injected intravenously into lethally-irradiated CD45.1 recipient mice. For LSK^+^s transplantation assay, 1000 freshly isolated LSK^+^s from *Setd5^CKO^* or *Setd5^fl/fl^* along with 1×10^6^ CD45.1 BM competitor cells were injected intravenously into lethally irradiated CD45.1 recipient. For the serial competitive transplantation assay, the same numbers of BM cells from recipients were pooled, and 2×10^6^ BM cells were then injected into lethally irradiated CD45.1 congenic recipients. For homing assay. BM cells from *Setd5^fl/fl^* and *Setd5^CKO^* mice were labeled with 5 mM CFSE (Thermo fisher) 3.5×10^6^ CFSE-labeled BM cells were transplanted into lethally irradiated CD45.2 mice. After 18 hours, CFSE^+^ cells in BM were analyzed with flow cytometry (Canto II, BD).

**RNA extraction and quantitative real-time polymerase chain reaction (qRT-PCR)**. Total RNA from BM samples or SLAM-HSCs was extracted using TRIzol™ Reagent (ThermoFisher) according to the manufacturer’s instructions. First-strand cDNA was synthesized with PrimeScript RT reagent Kit with gDNA Eraser (TAKARA) according to the manufacturer’s instructions. qRT-PCR was performed using Fast SYBR Green Master Mix (Roche) on QuantStudio 5 real-time PCR detector (ThermoFisher). All primers used in this study are listed in Supplemental Table S1.

**SLAM-HSCs RNA-sequencing and data analysis.** c-Kit^+^ BM cells were enriched with CD117 magnetic Beads (Miltenyi), and stained with Lin cocktail, Sca-1, c-Kit, CD150, CD48 antibodies. SLAM-HSCs were sorted with Aria III (BD). RNA was extracted using TRIzol reagent and amplified for 8 cycles with MALBAC Platinum Single RNA Amplification Kit (YIKON GENOMICS). RNA-seq libraries were prepared by using the NEBNext UltraTM RNA Library Prep Kit for Illumina (NEB) following manufacturer’s recommendations. RNA-seq libraries were sequenced by Illumina Hiseq platform and 150 bp paired-end reads were generated.

Low quality, adapter-containing and ploy-N containing reads were removed to obtain clean data (clean reads). The clean reads were mapped to the reference genome mm10 with Hisat2 (v2.0.5) [4]. FeatureCounts (v1.5.0-p3) [5] was used to count the reads numbers mapped to each gene. Differential gene expression analysis was performed with DESeq2 software [6]. DEGs were listed in Supplemental Table S2 and Table S3. Gene Set Enrichment Analyses (GSEA) was performed using GSEA software [7] (<http://software.broadinstitute.org/gsea/index.jsp>).

**Cell sorting and single cell RNA-sequencing**. Single-cell suspensions were prepared by collecting BM cells from 2 pairs of *Setd5^fl/fl^* and *Setd5^CKO^* mice. c-Kit^+^ cells were then enriched with CD117 magnetic beads, mixed for each group and stained with Lin cocktail, Sca-1, c-kit, CD150, CD48 antibodies. LSK^+^ cells were sorted directly into a 96-well PCR plate containing 2.55 μl of primer/lysis mix using the single-cell mode of a BD Arial III. For the scRNA-seq data generated in Figure 4, we used 92 indexed polydT primers with an 8N unique molecular identifier (UMI) sequence followed by an 8-bp index sequence. The reverse transcription and template switch steps were then performed following the Smart-seq2 protocol [8, 9]. Amplified complementary DNA was sonicated to ~300-bp fragments using the Covaris S2 system. The 3′ ends of the transcripts were enriched using Dynabeads Myone streptavidin C1 beads (Invitrogen) after biotin-primer PCR. The cDNA libraries were generated using a KAPA Hyper Prep kit for Illumina (KAPA) and sequenced on an Illumina Hiseq platform (Novogene).

**Primary process and quality control of scRNA-seq data**

We removed poly-A tails, template switching oligos (TSOs) sequences and adapters with Cutadapt (v3.0) [10] and trimmed low-quality bases with Trimmomatic (v0.39) [11]. Raw sequencing reads were demultiplexed and assigned to each single cell according to the unique barcode sequences pooled in sequencing libraries using code in Github (https://github.com/tallulandrews/scRNASeqPipeline). Sequencing reads were aligned to the GENCODE mouse reference genome (GRCm38.p6) using STAR (v2.7.6a) [12]. HTSeq (v0.12.4) [13] was used to summarize the uniquely aligned reads for each gene. Duplicated reads of transcripts were removed by referring to UMI sequences. The number of transcripts for each gene was calculated as the number of different UMIs detected. Low-quality cells were further removed using Scater R package (v1.18.3) [14]. In detail, the criteria for low-quality cells followed that the number of expressed genes or transcripts and the percentage of mitochondrial gene transcripts were deviated 3 times from the median value. Genes were kept for further analysis only if they were expressed in more than two cells. In total, we sequenced 1380 single cells and retained 1262 cells after the quality-control process of the primary sequencing data, including 585 cells for *Setd5^fl/fl^* mice and 677 for *Setd5^CKO^* mice. UMIs were normalized with the Scran R packages (v1.18.0) [15]. These processed data were then used as input to the Seurat (v3.0) [16] for variable genes finding, data integration and cell cycle effect regressing out.

**Heterogeneity and classification of hematopoietic cell types**

PCA (Principal Component Analysis) of single-cell gene expression profiles was performed on the top 2000 ranking variable genes. Then we used unsupervised hierarchical clustering analysis to group cells with similar expression patterns based on the top 24 PCs. Pheatmap (v1.0.12) (<https://github.com/raivokolde/pheatmap>) was used to generate a heat map. Marker genes for each cluster were identified by the FindAllMarkers functions and then ranked by p-value. The Sonia Nestorowa *et al*. data was downloaded and Spearman’s association test was performed between our single cell population and stem/progenitor cell population in Sonia Nestorowa *et al*. article with SingleR R packages (v1.4.0) [17], the Single R score was used as an indicator of the similarity between our single cell data and the Sonia Nestorowa *et al*. data. Due to limited space for the heat map in Figure S5G, the representing markers were selected for clusters. The complete list of marker genes was in Table S4.

**Enrichment analysis and cell cycle measurement**

Gene Set Enrichment Analyses was performed as previously described. Gene sets, including LT-HSC signature, proliferation, quiescence, myeloid, erythroid and lymphoid were obtained from published data [18-21]. GO term enrichment and KEGG analysis were performed using the clusterProfiler R package (v3.18.0) [22]. Differential expressed genes required the expression fold-change to be no less than 2 and the *P* value to be less than 0.05. The terms with adjusted *P* values of less than 0.1 were considered as significant enrichments. We assigned a cell cycle phase (G0/G1, S or G2/M) to each single cell using the ‘cyclone’ function in the Scran R package (v1.18.0) [15].

**Co-Immunoprecipitation and mass spectrometry**. SETD5-FLAG expressing stable MEL or Hela cell lines were lysed in IP lysis buffer containing 1% NP40, 10% Glycerol, 135 mM NaCl, 20 mM Tris-HCl pH8.0, supplemented with protease inhibitor (Roche) and phosphatase inhibitor (Roche) as previously described [23]. Whole cell lysates were pre-cleared and incubated with indicated antibody overnight (O/N). Protein-A/G beads were added 2 h and washed for three times. The beads were boiled directly with 2×SDS loading buffer and visualized on SDS-PAGE. Immunoprecipitated proteins were visualized by silver staining with Silver Stain Kit (Thermo Fisher) according to the manufacturer’s instructions. Gel was cut and separated with protein sizes, immunoprecipitated proteins in liquid from Hela cells and gels from MEL cells were analyzed by mass spectrometry. Details for proteins identified with mass spectrometry were listed in Supplemental Table S5. The LC-MS/MS was performed on a mass spectrometer coupled to Easy-nLC 1000 (Thermo Fisher Scientific). MS data was acquired using a data-dependent top10 method dynamically choosing the most abundant precursor ions from the survey scan (300–1,800 m/z) for HCD fragmentation. MS/MS spectra were searched using MASCOT engine (Matrix Science, version 2.2) against Universal Protein (UniProt) database.

**Chromatin immunoprecipitation (ChIP) assay**

Enriched c-Kit^+^ BM cells from *Setd5^fl/fl^* and *Setd5^CKO^* mice were cross-linked with a final concentration of 1% formaldehyde for 10 min at RT, followed by addition of glycine to a final concentration of 0.125 M. After wash, cells were suspended in ChIP lysis buffer (50 mM Tris-HCl pH 7.4, 1% SDS, 10 mM EDTA) containing freshly added 1× proteinase inhibitor cocktail and sonicated to generate DNA fragments of 150-400 bps with Covaris S220 sonicator (50 cycles of 230 s, 20 % amplitude, 300 watts). The soluble chromatin was diluted with dilution buffer (about 3×10^6^ cells per ml) and incubated O/N 4^o^C using 2 μg ChIP-grade antibodies. After overnight incubation, chromatin was incubated with 20 μl protein A/G magnetic beads (Invitrogen) for 2 hours, then washed twice sequentially with low salt buffer (50 mM HEPES pH 7.9, 1 mM EDTA, 1% EDTA, 0.1% SDS, 140 mM NaCl, 0.1% deoxycholate), high salt buffer (50 mM HEPES pH 7.9, 1 mM EDTA, 1% Triton X-100, 0.1% SDS, 500 mM NaCl, 0.1% deoxycholate), LiCl buffer (20 mM Tris-HCl pH8.0, 1 mM EDTA, 250 mM LiCl, 0.5% deoxycholate, 0.5% NP40) and TE buffer. The ChIP enriched DNA was eluted, reverse-crosslinked and treated with RNase and Protease K by standard ChIP assay protocol. DNA was extracted using phenol chloroform and ethanol precipitation.

**ChIP-sequencing (ChIP-seq) and ChIP-qPCR**. ChIP assays were done as described previously [24]. Details for antibodies and reagents were listed in Supplemental Table S1. Pol II, Pol II Ser2P, PAF1, H3K4me3, H3K9me2 and H3K36me3 ChIP assays were performed with enriched c-Kit^+^ BM cells. SETD5-FLAG ChIP was performed with SETD5-FLAG stably expressed MEL cell line. ChIP eluted DNA was used for qPCR or subjected to library construction for ChIP-seq (Novogene). The ChIP DNA library was constructed using the NEBNext ChIP-seq Library Prep Master Mix Set for Illumina. Library quality was assessed on the Agilent Bioanalyzer 2100. Pair-end sequencing of samples was performed on Illumina platform (Novogene).

Raw data was processed using fastp software [25] to removing low quality reads. Q20, Q30 and GC content of the clean data were calculated. All the downstream analyses were based on the clean data with high quality. Clean reads were aligned to the reference genome using Bowtie 2 (v2.3.5.1) [26]. Visualization of signal were used with deepTools (v3.4.3) [27]. We used The MACS2 (v2.1.4) [28] and epic2 (v0.0.43) [29] to identify regions of ChIP enrichment over background. A q-value threshold of 0.05 was used for all data sets. ChIPseeker [30] was used to retrieve the nearest genes around the peak and annotated genomic regions of the peaks, IGV (v2.7.2) [31] was used to visualize the ChIP-seq peaks. Pausing index were calculated by taking the average coverage of the promoter region (100 upstream of the TSS to 300 downstream of the TSS) divided by the average coverage of the gene body (300bp downstream of the TSS to 2kb downstream of the TSS).

**Statistical analysis**. All experiments were repeated two or three time with the indicated sample numbers. Sample size for each experiment was determined according to experience or the previously published papers. The investigator was not blinded during the experiment or assessing the outcome. Distribution was tested by Shapiro-Wilk normality test. When parameters followed Gaussian distribution, Student’s *t* test was used to compare two groups, One-way-ANOVA was used when comparing more than two groups, otherwise the Mann-Whitney test was employed. The variance is similar between the groups that are being statistically compared. Data were analyzed using Prism 8.0 (GraphPad Software). Poisson distribution-based analysis was used to calculate the functional HSCs frequencies in the limiting dilution experiments. In the figures, data are expressed as mean ± standard error (s.e.m) and significance was set at *P* < 0.05 (asterisks indicate * *P* < 0.05, ** *P* < 0.01, and *** *P* < 0.001). Sample size ‘n’ indicates biological replicates.


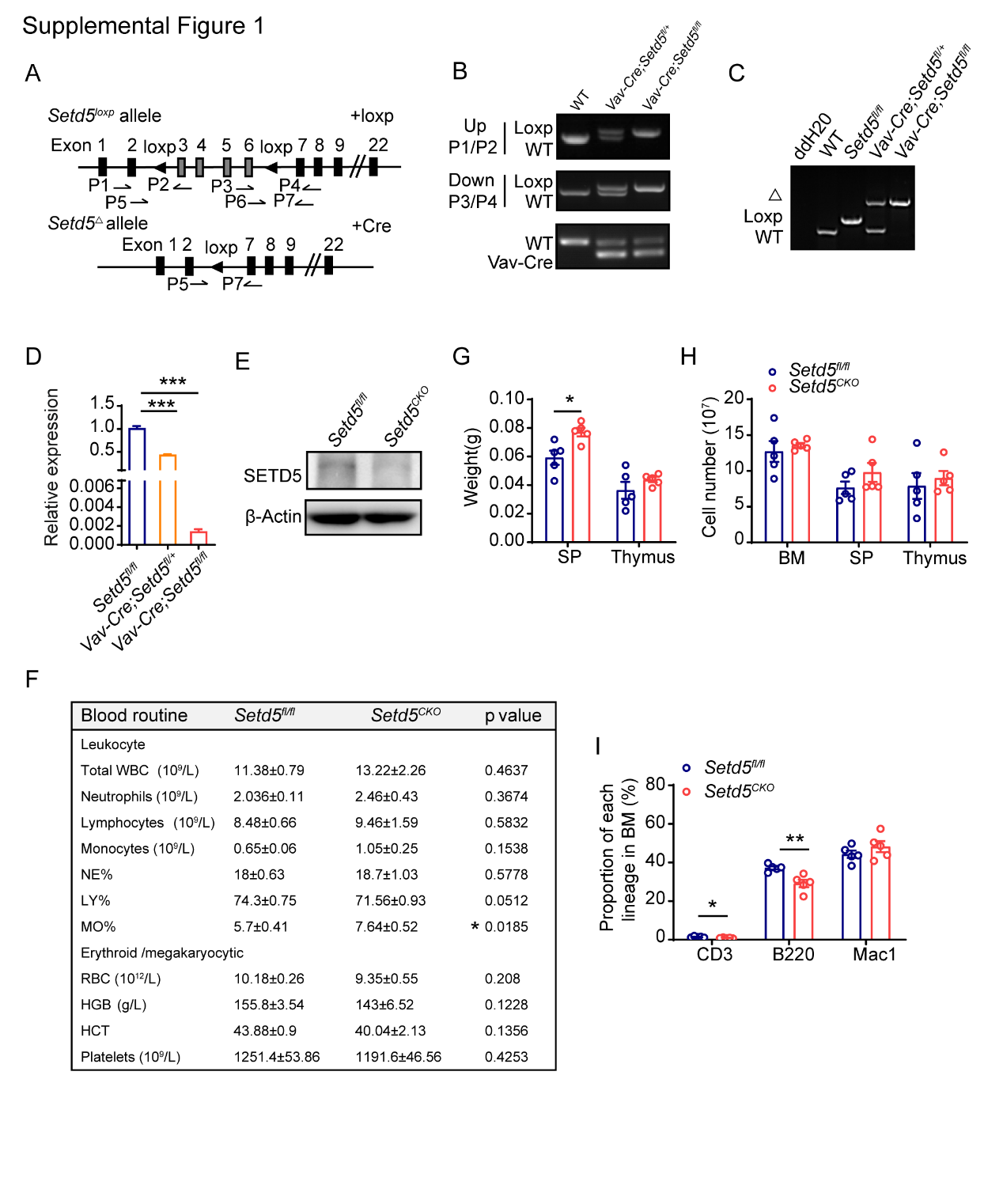


**Supplemental Figure 1. Deletion of *Setd5* in hematopoietic system affected hematopoietic homeostasis modestly.** **A** Targeting strategy to generate the *Setd5* conditional allele. Arrows: indicated the location of genotyping primers. **B** Representative PCR to detect the *Setd5^lox^* allele and *Vav-Cre* (P1:P2 for upper loxp site, P3:P4 for lower loxp site). **C** PCR of genomic DNA showed excision of the loxp allele upon *Vav-Cre* expression (primers P5:P7 and P6:P7 for ∆ allele). **D** Relative expression of *Setd5* in murine mature peripheral blood (PB) cells normalized to 18s; n=3. **E** Western blotting analysis of *Setd5* deletion in BM cells. **F** PB cell counts in 8-week-old *Setd5^fl/fl^* and *Setd5^CKO^* mice; n=5. **G** The weight of SP and thymus of animals; n=5. **H** The cell number of BM, SP and thymus. Femur, tibia and fibula were calculated for BM cellularity; n=5. **I** FACS analysis the frequencies of T, B, and myeloid cells in BM cells; n=5. Mean ± SEM. * *P* < 0.05, ** *P* < 0.01.


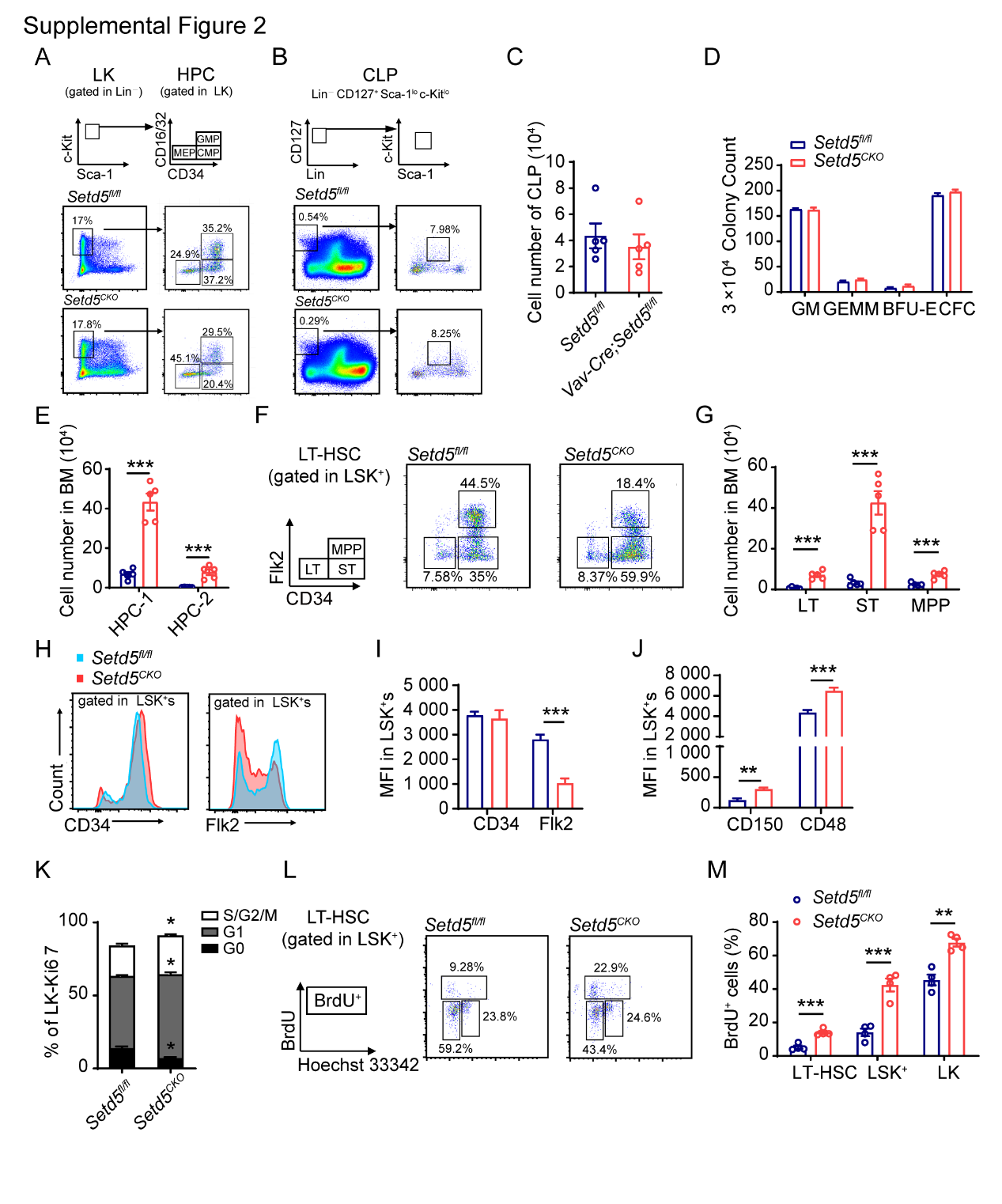


**Supplemental Figure 2. *Setd5* deficiency altered HSC frequency and led to LK expansion.** **A-B** FACS gating strategy for HPCs and CLPs in BM; n=5. **C** The absolute cell number of CLP cells; n=5. **D** Colony forming assay of BM cells from *Setd5^fl/fl^* and *Setd5^CKO^* mice; n=3. **E** The absolute cell number HPC-1 and HPC-2 cells in BM; n=5. **F-G** FACS analysis and absolute cell number of LT-HSC, ST-HSC and MPP in BM; n=5. **H-J** MFI (median) values of CD34, Flk2, CD150 and CD48; n=5. **K** Ki67 staining of LKS^–^ cells in *Setd5^fl/fl^* and *Setd5^CKO^* mice, Representative FACS profiles and the frequency of G0, G1, S/G2/M cells are shown; n=4. **L-M** BrdU incorporation analysis of LT-HSC and LK cells in *Setd5^fl/fl^* and *Setd5^CKO^* mice; n=4. Mean ± SEM, * *P* < 0.05; ** *P* < 0.01.


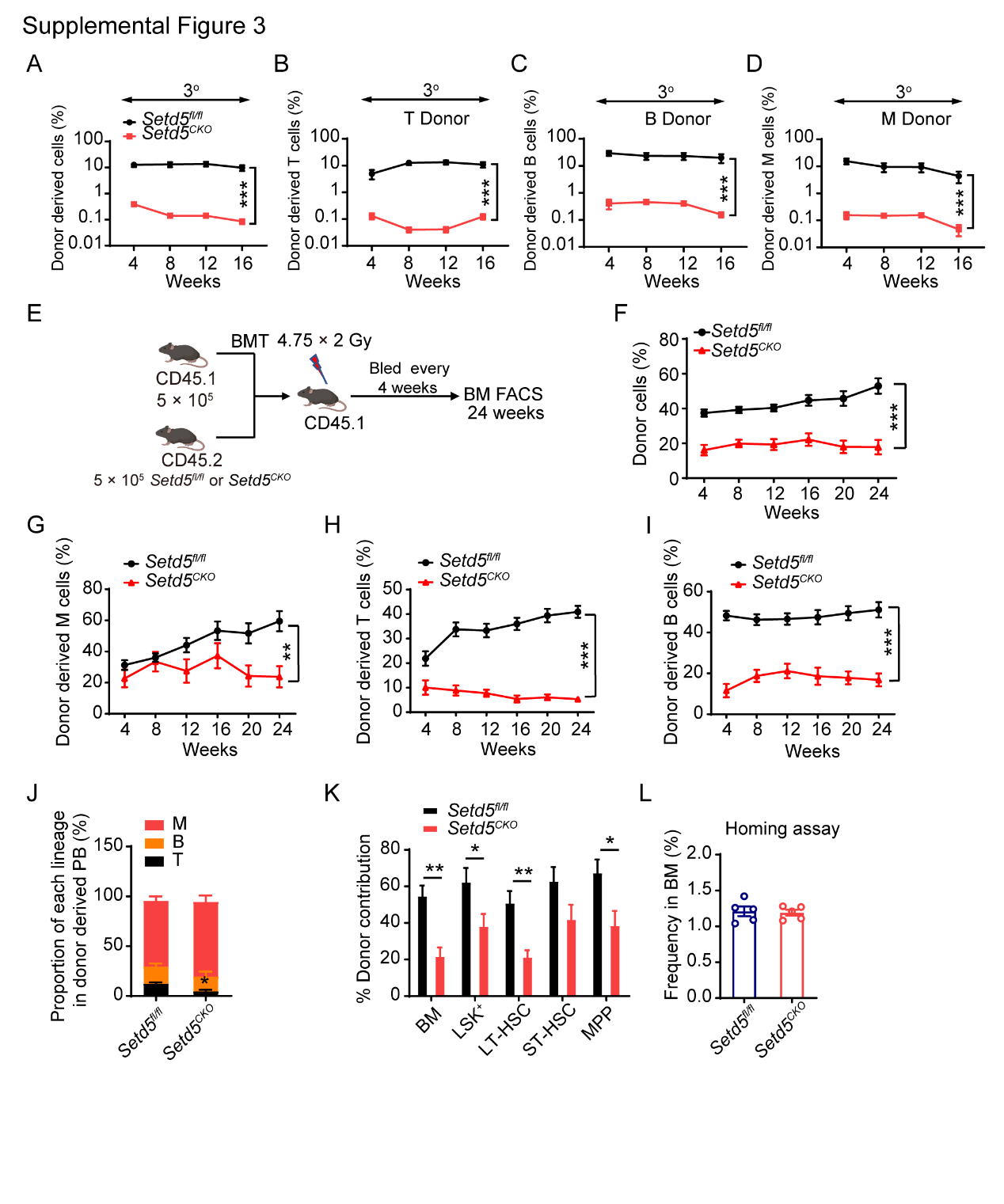


**Supplemental Figure 3. BM competitive transplantation assay revealed that** ***Setd5^CKO^* HSCs have a decreased lymphoid repopulation capacity.** **A-D** Percentage of donor-derived cells, T cells, B cells, and myeloid cells in the tertiary LSK^+^ competitive transplantation assay, n=8 or 9. **E** Strategy for competitive repopulation assay with *Setd5^fl/fl^* or *Setd5^CKO^* BM cells, n=11. **F-I** Percentage of donor-derived cells, T cells, B cells, and myeloid cells in the PB of recipient animals 24 weeks after transplantation, respectively; n=11 or 6. **J** Proportion of each lineage in donor derived PB cells; n=5. **K** Donor contribution of indicated cell populations in BM cells of recipient mice at 24 weeks; n=5. **L** Homing assay: Percentage of CD45.2 cells in the BM 18 hours after injection of 3.5×10^6^ CFSE^+^ BM cells into irradiated CD45.1 mice; n=5. Mean ± SEM, * *P* < 0.05, ** *P* < 0.01, *** *P* < 0.001.


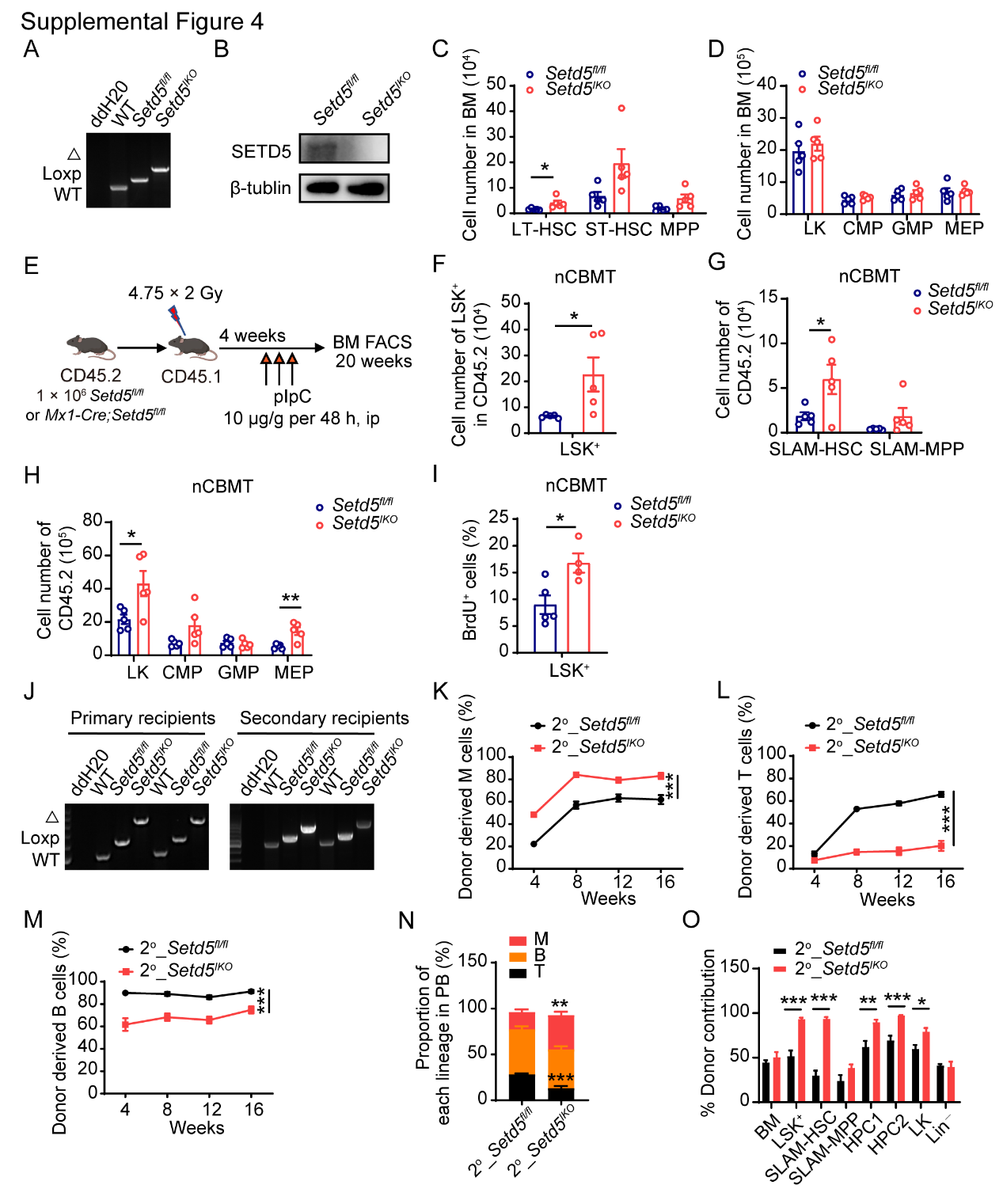


**Supplemental Figure 4. *Setd5* exerted an intrinsic regulatory role in HSCs. A** PCR of genomic DNA showed excision of the loxp allele upon *Mx1-Cre* expression in BM cells. **B** Western blotting analysis of *Setd5* deletion in BM cells upon *Mx1-Cre* expression. **C** Relative frequency of LT-HSC subsets in BM cells from *Setd5^fl/fl^* and *Setd5^IKO^* mice; n=5. **D** The absolute number of HPC cells in *Setd5^fl/fl^* and *Setd5^IKO^* mice; n=5. **E** Schematic diagram for non-competitive transplantation assay with *Setd5^fl/fl^* and *Mx1*-*Cre*; *Setd5^fl/fl^* BM cells. **F-H** The absolute number of HSPC populations in *Setd5^fl/fl^* and *Setd5^IKO^* recipient mice; n=5. **I** Cell cycle analysis of LSK^+^s in *Setd5^fl/fl^* and *Setd5^IKO^* recipients; *Setd5^fl/fl^*=5, *Setd5^IKO^*=4. **J** PCR of genomic DNA showed excision of the loxp allele upon *Mx1-Cre* expression in primary and secondary recipients. **K-M** Percentage of donor-derived T cells, B cells and myeloid cells in the PB of *Setd5^fl/fl^* and *Setd5^IKO^* secondary recipient mice at the indicated time points; n=9. **N** Proportion of each lineage in donor derived PB cells from secondary recipient mice; n=5. **O** Donor contribution of indicated cell populations in BM cells of secondary recipient mice; n=5. Mean ± SEM, * *P* < 0.05; ** *P* < 0.01.


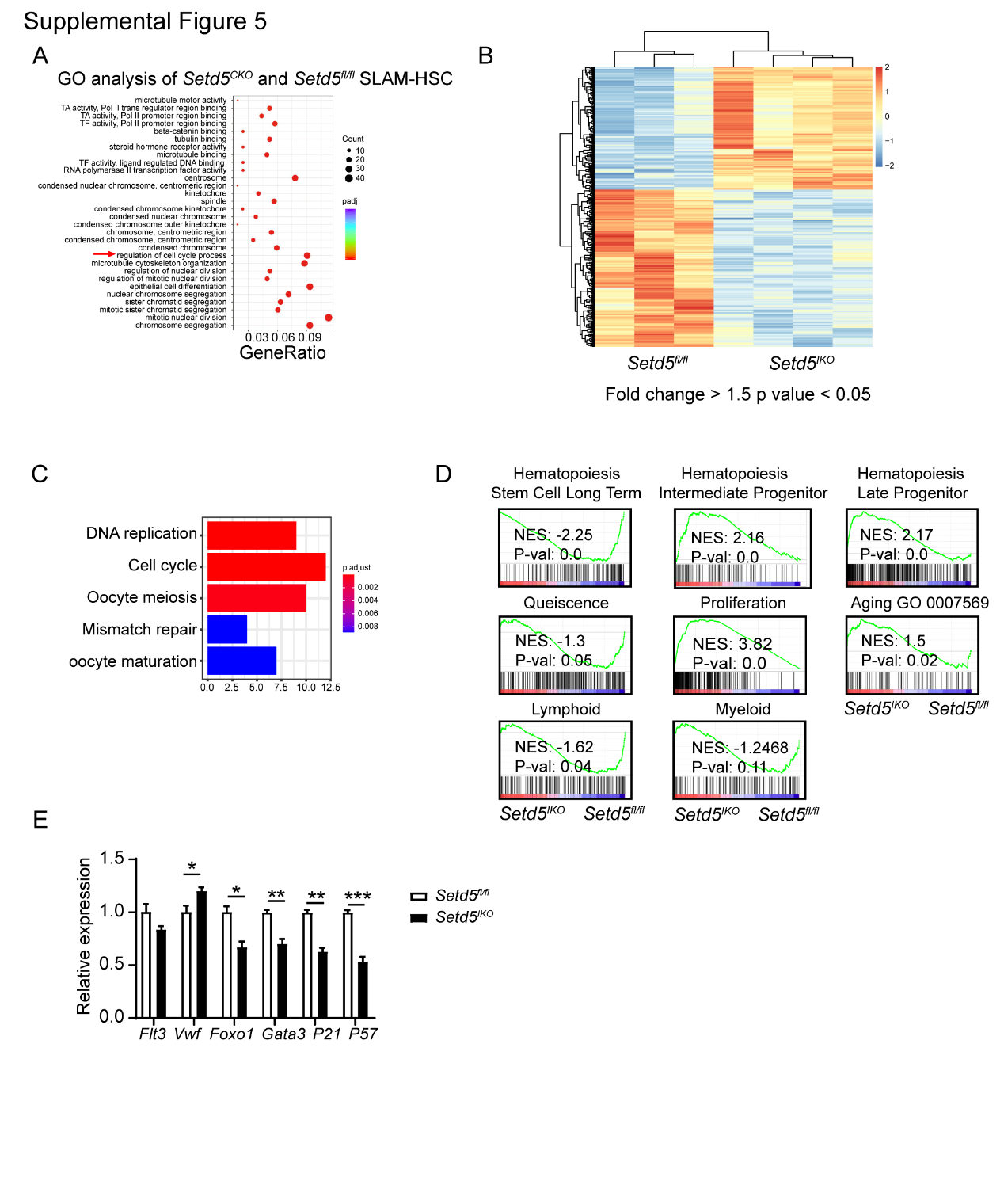


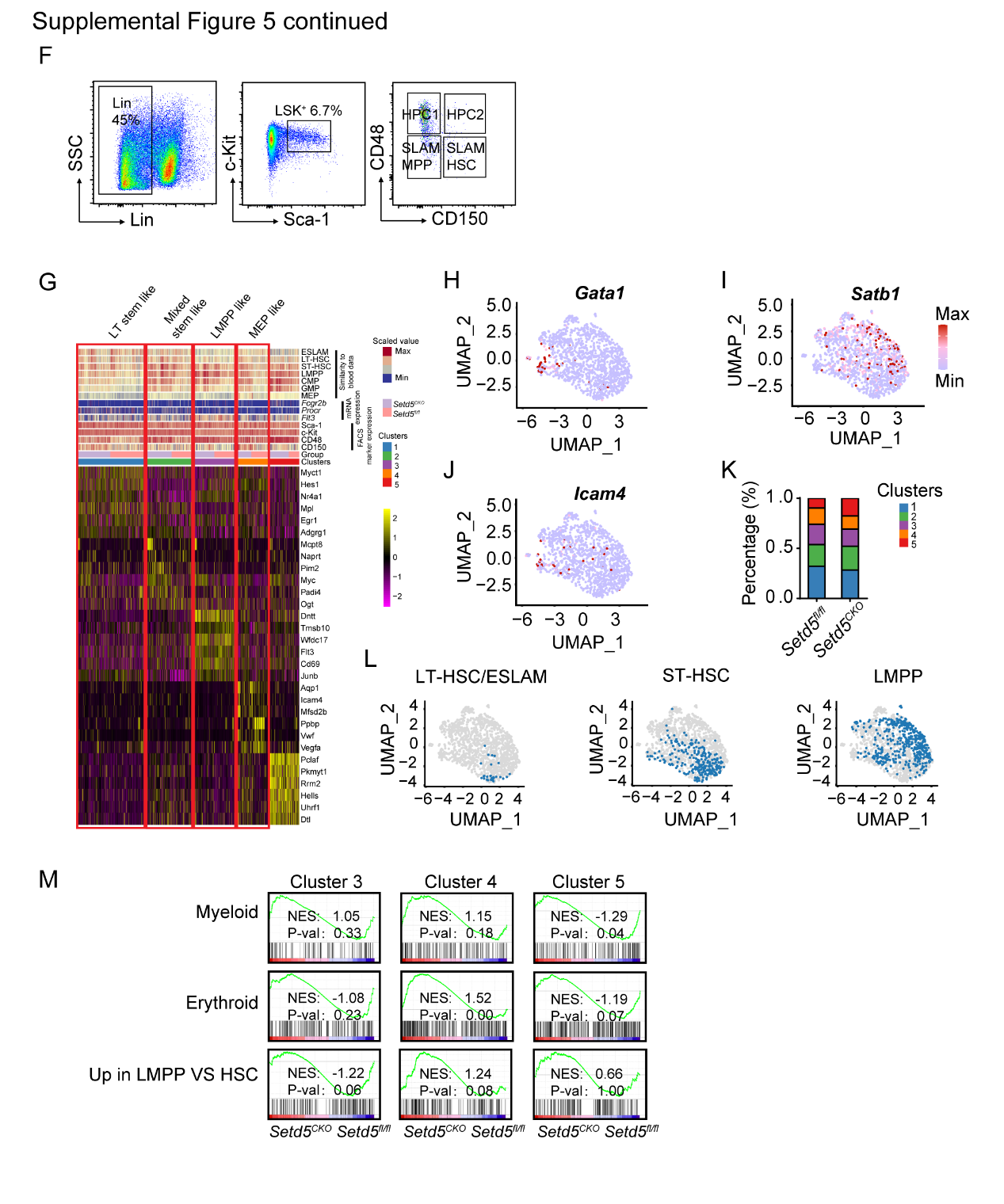


**Supplemental Figure 5. *Setd5* depletion altered stem cell property and lineage commitment.** **A** Gene ontology analysis of differentially expressed genes in *Setd5^CKO^* SLAM-HSC. **B** Heatmap of 330 DEGs in *Setd5^fl/fl^* and *Setd5^IKO^* SLAM-HSC. Fold change > 1.5, p value < 0.05. **C** Enrichment analysis of the DEGs in *Setd5^IKO^* SLAM-HSC. **D** GSEA analyses for genes affected in the SLAM-HSCs of *Setd5^fl/fl^* and *Setd5^IKO^* mice. NES, normalized enrichment score. **E** Relative expression levels of cell cycle and multi-potency genes in HSC cells; n=3. mRNA levels were normalized to the expression of 18s. **F** Gating strategy for single cell sorting of LSK^+^ cells. **G** Heat map of signature gene expression for each cluster identified using Seurat. A Similarity score to Nestorowa *et al*. data, classic marker gene expression level and index sorted FACS marker levels are annotated on top. Cluster 1 (LT stem-like), cluster 2 (Mixed stem-like) cluster 3 (LMPP-like) and cluster 4 (MEP-like) are framed. **H-J** Diffusion map of all cells was colored according to the expression of selected genes and the color corresponds to a log2 scale of expression ranging between 0 and the maximum value for each gene. **K** Histograms showing the compositions of transcriptome-defined five clusters in two groups. **L** We annotated transcriptome-defined 5 clusters of LSK^+^s with cell types defined in Nestorowa *et al*. paper. Diffusion map of LT-HSC, ST-HSC and LMPP was colored. **M** GSEA analysis of genes associated with myeloid and erythroid signatures in cluster 3-5, as well as genes upregulated in LMPP compared with HSC.


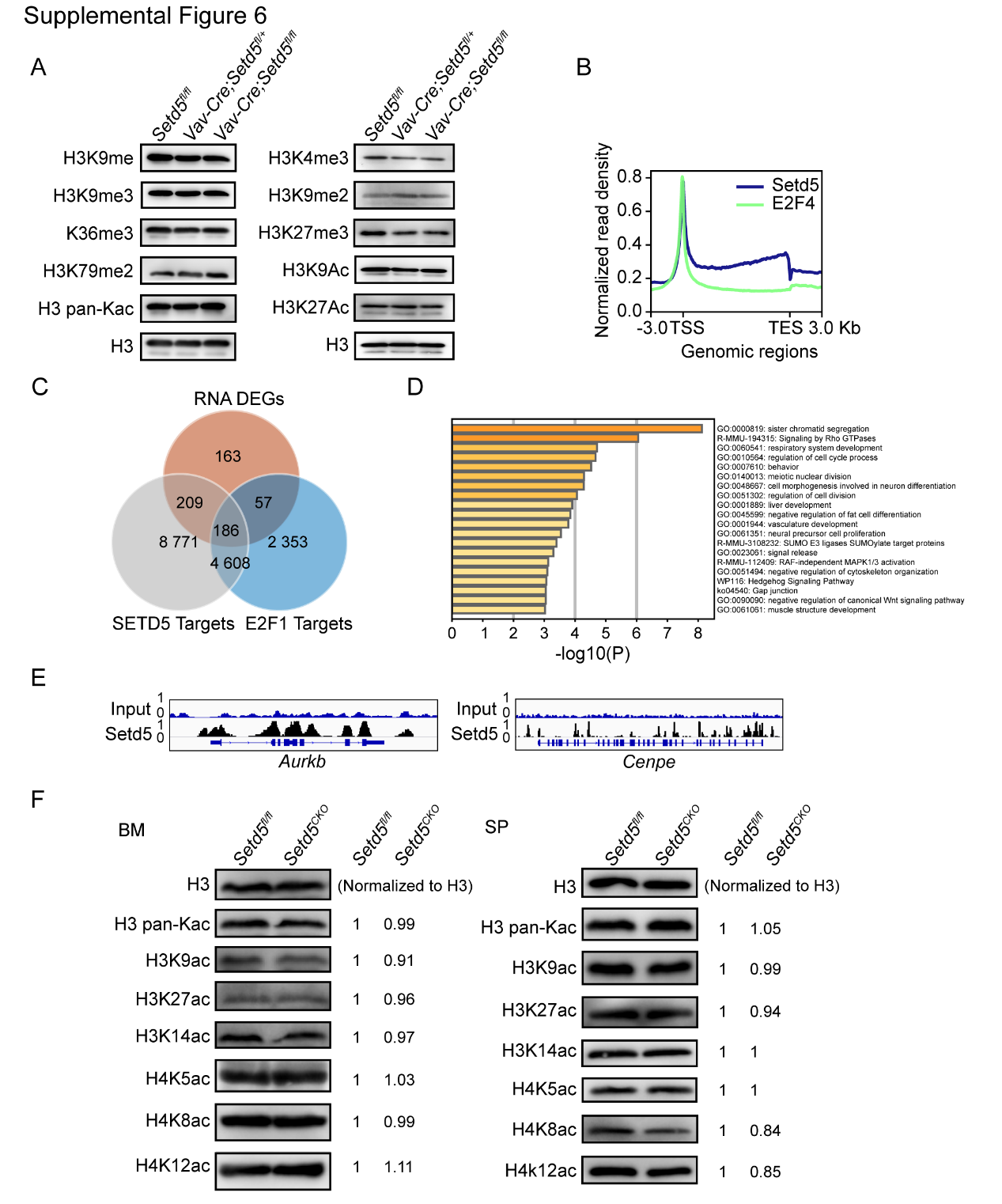


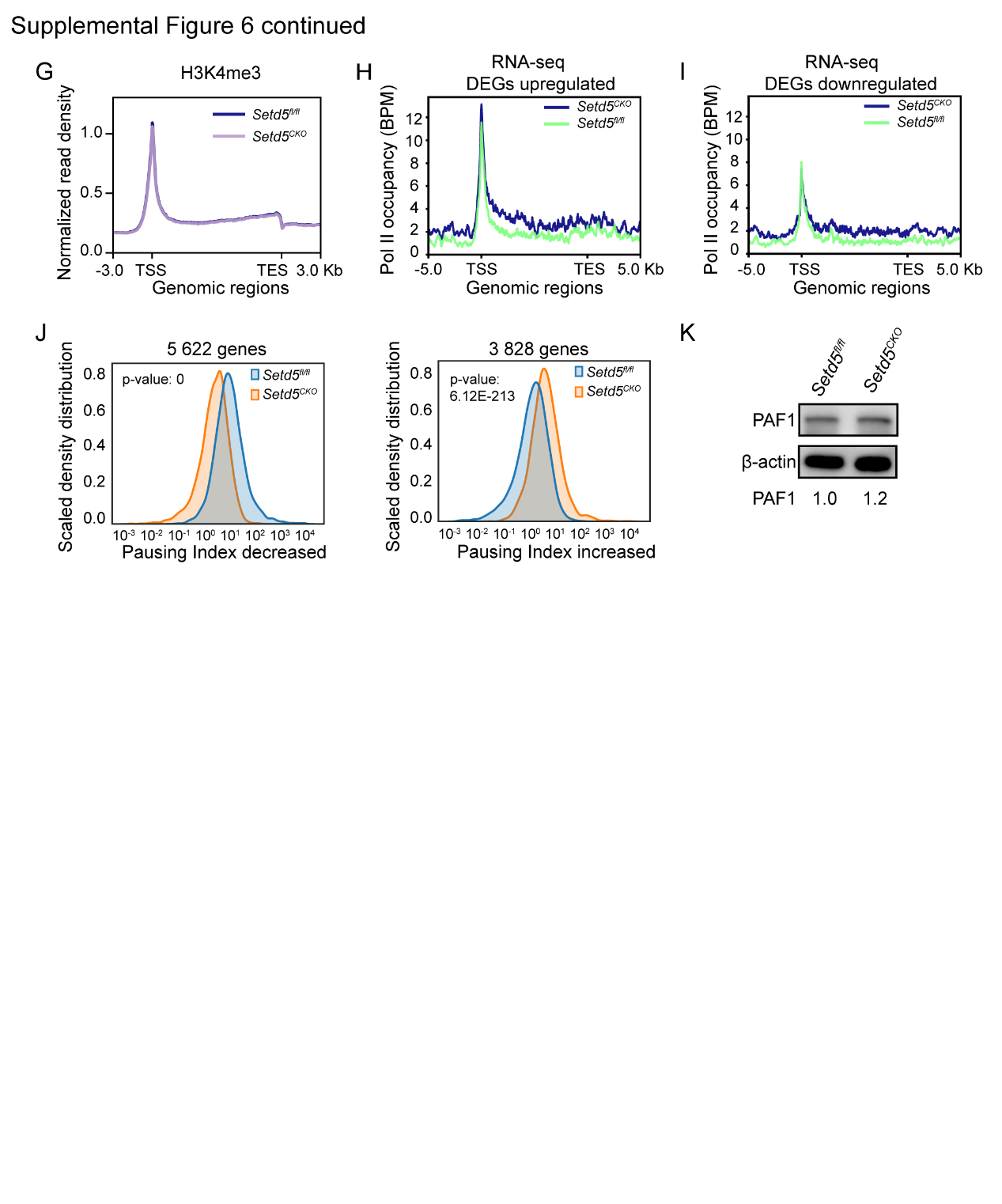


**Supplemental Figure 6. SETD5 deficiency did not affect histone methylation and acetylation level but promoted the promoter-proximal paused Pol II release.** **A** Western blotting analysis of protein lysates from *Setd5^fl/fl^* and *Setd5^CKO^* BM cells probed with antibodies against the indicated histone modifications respectively, as well as histone H3 as a loading control. **B** Density plots of SETD5 (blue) and E2F4 (green) normalized ChIP-seq signals. **C-D** Venn diagrams showing the overlap between SETD5 targets, E2F1 targets and DEGs, with enrichment analysis of the common genes (186 genes) by Metascape [32]. **E** IGV tracks of indicated genes for SETD5 occupancy in MEL cells. **F** Immunoblot analysis of protein lysates from *Setd5^fl/fl^* and *Setd5^CKO^* c-Kit^+^ BM or SP cells. Histone H3 is as a loading control, and densitometry measurement was normalized to H3 per group and then normalized to *Setd5^fl/fl^* group. **G** Density plot of H3K4me3 in *Setd5^fl/fl^* and *Setd5^CKO^* c-Kit^+^ cells. **H-I** Occupancy of Pol II on upregulated-DEGs and downregulated-DEGs (Fold change>1.5 and p adj value<0.05), ChIP normalization was implemented by integrating Spike-in Chromatin. **J** Density plot of genes with reduced (left, n=5622) or increased (right, n=3828) Pol II pausing index in *Setd5^CKO^*. **K** Immunoblot analysis of PAF1 in c-Kit^+^ cells.


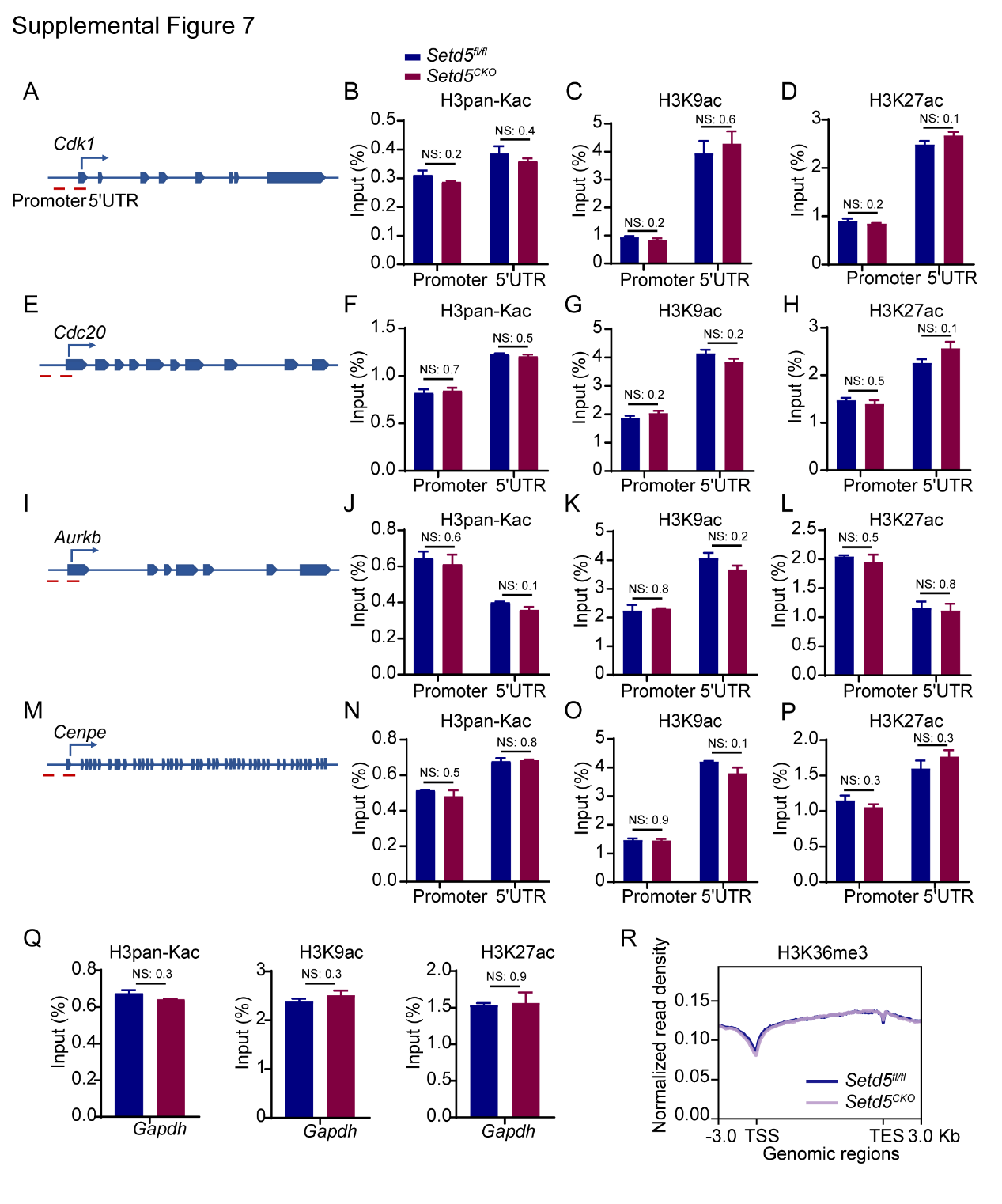


**Supplemental Figure 7. The effect of SETD5 on Pol II pausing was independent of SETD5-HDAC3/NCOR complexes. A** Schematic of the mouse *Cdk1* gene locus showing the location of ChIP-qPCR primers. **B-D** H3 pan-Kac, H3K9ac and H3K27ac ChIP analysis of *Cdk1* gene locus in *Setd5^fl/fl^* and *Setd5^CKO^* c-Kit^+^ BM cells; n=3. **E** Schematic of the mouse *Cdc20* locus and the location of primers. **F-H** H3 pan-Kac, H3K9ac and H3K27ac ChIP analysis of *Cdc20* gene locus; n=3. **I** Schematic of the mouse *Aurkb* locus and the location of primers. **J-L** H3 pan-Kac, H3K9ac and H3K27ac ChIP analysis of *Aurkb* gene locus; n=3. **M** The *Cenpe* locus and the location of primers. **N-P** H3 pan-Kac, H3K9ac and H3K27ac ChIP analysis of *Cenpe* gene locus; n=3. **Q** H3 pan-Kac, H3K9ac and H3K27ac ChIP analysis of *Gapdh*. *Gapdh* is an internal control; n=3. **R** Density plot of H3K36me3 in *Setd5^fl/fl^* and *Setd5^CKO^* c-Kit^+^ cells.

**References**

1. Guo H, Chu Y, Wang L, Chen X, Chen Y, Cheng H*, et al.* PBX3 is essential for leukemia stem cell maintenance in MLL-rearranged leukemia. Int J Cancer*.* 2017; 141**:** 324-35.

2. Wang X, Gao Y, Gao J, Li M, Zhou M, Wang J*, et al.* Rheb1 loss leads to increased hematopoietic stem cell proliferation and myeloid-biased differentiation in vivo. Haematologica*.* 2018.

3. Chu Y, Chen Y, Li M, Shi D, Wang B, Lian Y*, et al.* Six1 regulates leukemia stem cell maintenance in acute myeloid leukemia. Cancer Sci*.* 2019; 110**:** 2200-10.

4. Kim D, Langmead B, Salzberg SL. HISAT: a fast spliced aligner with low memory requirements. Nat Methods*.* 2015; 12**:** 357-60.

5. Liao Y, Smyth GK, Shi W. featureCounts: an efficient general purpose program for assigning sequence reads to genomic features. Bioinformatics*.* 2014; 30**:** 923-30.

6. Love MI, Huber W, Anders S. Moderated estimation of fold change and dispersion for RNA-seq data with DESeq2. Genome Biol*.* 2014; 15**:** 550.

7. Subramanian A, Tamayo P, Mootha VK, Mukherjee S, Ebert BL, Gillette MA*, et al.* Gene set enrichment analysis: a knowledge-based approach for interpreting genome-wide expression profiles. Proc Natl Acad Sci U S A*.* 2005; 102**:** 15545-50.

8. Li L, Dong J, Yan L, Yong J, Liu X, Hu Y*, et al.* Single-Cell RNA-Seq Analysis Maps Development of Human Germline Cells and Gonadal Niche Interactions. Cell Stem Cell*.* 2017; 20**:** 858-73.e4.

9. Picelli S, Faridani OR, Björklund AK, Winberg G, Sagasser S, Sandberg R. Full-length RNA-seq from single cells using Smart-seq2. Nat Protoc*.* 2014; 9**:** 171-81.

10. Martin M. Cutadapt removes adapter sequences from high-throughput sequencing reads. EMBnetjournal; Vol 17, No 1: Next Generation Sequencing Data AnalysisDO - 1014806/ej171200*.* 2011.

11. Bolger AM, Lohse M, Usadel B. Trimmomatic: a flexible trimmer for Illumina sequence data. Bioinformatics*.* 2014; 30**:** 2114-20.

12. Dobin A, Davis CA, Schlesinger F, Drenkow J, Zaleski C, Jha S*, et al.* STAR: ultrafast universal RNA-seq aligner. Bioinformatics*.* 2013; 29**:** 15-21.

13. Anders S, Pyl PT, Huber W. HTSeq--a Python framework to work with high-throughput sequencing data. Bioinformatics*.* 2015; 31**:** 166-9.

14. McCarthy DJ, Campbell KR, Lun AT, Wills QF. Scater: pre-processing, quality control, normalization and visualization of single-cell RNA-seq data in R. Bioinformatics*.* 2017; 33**:** 1179-86.

15. Lun AT, McCarthy DJ, Marioni JC. A step-by-step workflow for low-level analysis of single-cell RNA-seq data with Bioconductor. F1000Res*.* 2016; 5**:** 2122.

16. Stuart T, Butler A, Hoffman P, Hafemeister C, Papalexi E, Mauck WM, 3rd*, et al.* Comprehensive Integration of Single-Cell Data. Cell*.* 2019; 177**:** 1888-902.e21.

17. Aran D, Looney AP, Liu L, Wu E, Fong V, Hsu A*, et al.* Reference-based analysis of lung single-cell sequencing reveals a transitional profibrotic macrophage. Nat Immunol*.* 2019; 20**:** 163-72.

18. Cabezas-Wallscheid N, Buettner F, Sommerkamp P, Klimmeck D, Ladel L, Thalheimer FB*, et al.* Vitamin A-Retinoic Acid Signaling Regulates Hematopoietic Stem Cell Dormancy. Cell*.* 2017; 169**:** 807-23.e19.

19. Ng SY, Yoshida T, Zhang J, Georgopoulos K. Genome-wide lineage-specific transcriptional networks underscore Ikaros-dependent lymphoid priming in hematopoietic stem cells. Immunity*.* 2009; 30**:** 493-507.

20. Chambers SM, Boles NC, Lin KY, Tierney MP, Bowman TV, Bradfute SB*, et al.* Hematopoietic fingerprints: an expression database of stem cells and their progeny. Cell Stem Cell*.* 2007; 1**:** 578-91.

21. Venezia TA, Merchant AA, Ramos CA, Whitehouse NL, Young AS, Shaw CA*, et al.* Molecular signatures of proliferation and quiescence in hematopoietic stem cells. PLoS Biol*.* 2004; 2**:** e301.

22. Yu G, Wang LG, Han Y, He QY. clusterProfiler: an R package for comparing biological themes among gene clusters. Omics*.* 2012; 16**:** 284-7.

23. Chu Y, Zhao Z, Sant DW, Zhu G, Greenblatt SM, Liu L*, et al.* Tet2 Regulates Osteoclast Differentiation by Interacting with Runx1 and Maintaining Genomic 5-Hydroxymethylcytosine (5hmC). Genomics Proteomics Bioinformatics*.* 2018; 16**:** 172-86.

24. Chu Y, Chen Y, Guo H, Li M, Wang B, Shi D*, et al.* SUV39H1 regulates the progression of MLL-AF9-induced acute myeloid leukemia. Oncogene*.* 2020.

25. Chen S, Zhou Y, Chen Y, Gu J. fastp: an ultra-fast all-in-one FASTQ preprocessor. Bioinformatics*.* 2018; 34**:** i884-i90.

26. Langmead B, Salzberg SL. Fast gapped-read alignment with Bowtie 2. Nat Methods*.* 2012; 9**:** 357-9.

27. Ramírez F, Ryan DP, Grüning B, Bhardwaj V, Kilpert F, Richter AS*, et al.* deepTools2: a next generation web server for deep-sequencing data analysis. Nucleic Acids Res*.* 2016; 44**:** W160-5.

28. Zhang Y, Liu T, Meyer CA, Eeckhoute J, Johnson DS, Bernstein BE*, et al.* Model-based analysis of ChIP-Seq (MACS). Genome Biol*.* 2008; 9**:** R137.

29. Stovner EB, Sætrom P. epic2 efficiently finds diffuse domains in ChIP-seq data. Bioinformatics*.* 2019; 35**:** 4392-93.

30. Yu G, Wang LG, He QY. ChIPseeker: an R/Bioconductor package for ChIP peak annotation, comparison and visualization. Bioinformatics*.* 2015; 31**:** 2382-3.

31. Robinson JT, Thorvaldsdóttir H, Winckler W, Guttman M, Lander ES, Getz G*, et al.* Integrative genomics viewer. Nat Biotechnol*.* 2011; 29**:** 24-6.

32. Zhou Y, Zhou B, Pache L, Chang M, Khodabakhshi AH, Tanaseichuk O*, et al.* Metascape provides a biologist-oriented resource for the analysis of systems-level datasets. Nat Commun*.* 2019; 10**:** 1523.
